# Monitoring the safety status of service bolts in mining roadways

**DOI:** 10.1371/journal.pone.0267099

**Published:** 2022-04-28

**Authors:** Jianjun Dong, Zhengquan Xie, Gaoyang Zheng, Hao Jiang

**Affiliations:** 1 College of Safety Science and Engineering, Liaoning Technical University, Huludao, Liaoning, China; 2 Key Laboratory of Mine Thermodynamic Disasters and Control of Ministry of Education, Liaoning Technical University, Huludao, Liaoning, China; Istituto Italiano di Tecnologia, ITALY

## Abstract

To monitor the safety status of the bolts in coal mining roadways in real time, the safety and stability of the bolt support structure were evaluated. Based on the conventional support bolts used in the field, a fiber Bragg grating (FBG) sensor and medium materials were selected. Through theoretical analysis, the bolt tension, and FBG temperature tests, the strain transmission mechanism of the FBG bolt was analyzed, and it was ensured that the developed FBG bolt could accurately measure the strain of the bolt. In the field test, FBG bolts were arranged on the positive and negative sides of the mining roadway to accurately monitor the safety status of the bolts in service in real time, and the force characteristics of the bolts monitored by the FBG sensor were analyzed to obtain the maximum axial force of the positive and negative bolts. Thereafter, the safety status of the roadway bolt was evaluated. The results show that the positive side bolts axial force change is significantly greater than that of the negative side bolt; with the working face advancing to a distance of 60 m from the bolt as the dividing line, the positive side bolts axial force grows slowly before this, after which the axial force increases rapidly. The locations of the roadway where the positive and negative bolts are most affected by mining are determined, and roadway support and prevention measures for this location should be conducted in time. The safety status of the bolts is evaluated and monitored as follows: the positive side No. 2, No. 3, No. 5, and No. 6 bolts have reached the failure state, the positive side No. 4 bolt is in a dangerous state, the positive side No. 1, negative side No. 8 and No. 9 are in an abnormal state, and the negative side No. 7, No. 10, No. 11, and No. 12 are in a normal condition. This research has laid a technical foundation for the real-time monitoring of the bolt support of the mining roadway and the assessment of the safety status of bolts.

## Introduction

The stability of mining roadways is a crucial factor for safe mine production [1]. In mining roadways, high-precision real-time monitoring of the stress and strain of the supporting bolts is combined with the stress and deformation states at different positions under the influence of mining to obtain the supporting characteristics of the bolts, which is effective in reducing the occurrence of roof fall [2].

In recent years, fiber Bragg grating (FBG) sensing technology has been extensively researched and applied. Hong Chengyu (2021) et al. studied surface-adhesive bare fiber gratings, deduced the fiber and substrate layer’s strain transfer formula, and verified the correctness of the theory [3]. Ma Jiaxiao (2020) et al. explored the applicability of the FBG sensor to test the penetration characteristics of static pressure piles with different pile diameters. The results show that the FBG sensor has high linearity and sensitivity, directly reflecting the differences and change features of the model piles during the penetration process [4]. Choi SangJin (2021) et al. installed FBG strain sensors in 1 km deep boreholes to monitor deep underground fault behavior and improved the applicability of FBG sensors in deep borehole environments through protection and installation [5]. Liu (2021) et al. applied FBG sensing technology, combined with additive manufacturing technology, to develop sensors to monitor the internal pressure of the soil [6]. Mieloszyk Magdalena (2021) proposed a feasibility analysis using FBG sensors to detect fatigue cracks and monitor their expansion process [7]. It can be observed that the application range of FBG sensors is rapidly expanding, with obvious advantages of multiple parameters, scales, and dimensions [8].

Research on the safety and stability of working face roadway monitoring and support has been continuously developed and innovated. Xu Qingyun (2020) et al. analyzed the occurrence, development mechanism, and influencing factors of the surrounding loose rock zone of a roadway. They used the knowledge of elastoplastic mechanics to conduct theoretical research on the failure mechanism of surrounding rock in deep re-mining roadways [9]. Hou Dinggui (2019) designed constant resistance monitoring bolts, monitored the entire roadway deformation and instability process, and studied the judgment conditions of surrounding rock instability [10]. Dengyun (2017) et al. conducted roof separation observations and roadway roof subsidence observations on roadways supported by bolts and cables. They analyzed the critical value of roof separation for various roadways [11]. Peng Wang (2020) et al. used the principle of fiber grating sensing to develop and apply force-measuring bolts and systems to verify the feasibility of force-measuring bolts, which are used to monitor the pressure of coal mine roadways [12]. Hao Jian (2021) et al. analyzed the influence of active and passive supports on roadway stability. They proposed a support scheme to effectively control the deformation of the surrounding rock [13]. Jing Hongdi (2019) researched and developed a continuous convergence and deformation monitoring system for the mining roadway section, which can complete underground roadway convergence and deformation monitoring [14]. Danqi Li (2020, 2021) et al. built a constitutive model simulating the load-displacement performance of rock bolts under axial loading, who researched the mechanical behaviour of different bolts combining with experimental analysis to reveal the bolt failure mechanism effectively [15–18].

At present, among the numerous research results, there are several studies on fiber grating technology applied to roadway support bolts. Existing roadway-support monitoring technology fails to achieve real-time monitoring and accurately reflects the safety status of the roadway support. To improve the accuracy of monitoring the surrounding rock and reflect the stress and strain state of the roadway support bolt in real time, a self-developed distributed bolt FGB sensor is used to arrange in the bolt body to realize high-precision monitoring of the axial force distribution. Based on the indoor tensile test to verify the feasibility and factual accuracy of the distributed bolt FGB sensor, the monitoring data of the field test were analyzed to obtain the safety state of the supporting bolt of the mining roadway. Thereafter, the accurate position of the top bolt axial force is acquired such that mine roadway safety accidents can be effectively prevented.

## Layout plan for monitoring fiber Bragg grating sensors

### Basic situation of working face

The fully mechanized caving face of a coal mine in the Shendong mining area was arranged in the inclined direction of the coal seam. Along the strike direction of the coal seam, the inclined length of the working face was 320 m. The strike length is 4485.2 m. The ground elevation was 1251.9~1346.7 m, and the floor elevation was 897.42~948.74 m. The coal seam in the mining section has a depth of 390–410 m, a coal thickness of 5.6–6.2 m, and an inclination angle of 1–3°.

### Layout of the working face roadway

The fully mechanized longwall panel adopts a three-roadway layout, and three roadways along the channel are arranged. The transportation channel is a belt equipped with a retractable belt conveyor to transport coal. It is used as a passage for coal transportation and working-face personnel. The auxiliary transportation channel is also used as an auxiliary transportation lane for working face equipment and materials.

### Monitoring sensor layout

The layout of the FBG sensor bolt is as follows.

1) Through statistical analysis of the actual occurrence of bolt breakage in the well, the location of the bolt is determined with a high probability that the problem will occur.

It can be determined from the mine pressure report records of the working face that the bolt damage caused by the periodic roof pressure occurs in both the transport tunnel and the auxiliary transport tunnel. The specific locations are mainly concentrated on the positive and negative sides of the tunnel.

2) The monitoring range of the roadway and the arrangement number of FBG sensor bolts are determined in the test section.

According to the pre-work period, the maximum step distance is 15m, and the maximum distance of the monitoring section is determined to be 15m. Because there is no obvious difference in the failure of the bolts in the two parallel groove roadways of the working face, the most suitable monitoring test roadway can be selected due to the site conditions. 2 FBG sensor bolts are arranged in the positive and negative sides of each section, and a total of 12 FBG sensor bolts are required to be arranged.

## Designation of fiber Bragg grating bolt

### Working principle of fiber Bragg grating bolt sensor

The FBG sensor grating reflects the light emitted from the broadband light source and is guided by the fiber coupler. The adjustment device measures the change in the wavelength.

The FBG sensors meet Bragg conditions:

$$\lambda_{B}=2n_{eff}\Lambda ,$$ (1)

where *λ*_*B*_ is the central wavelength of the reflected light (nm), *n*_*eff*_ is the effective refractive index, and Λ is the grating period.

The relationship between temperature *T*, strain *ε*, and *λ*_*B*_ of the FBG sensor is:

$$\Delta \lambda_{B}=\lambda_{B}\{1-\frac{n_{eff}^{2}}{2}[p_{12}-\nu (p_{11}+p_{12})]\}\varepsilon +\lambda_{B}(\alpha +\xi )\Delta T,$$ (2)

where *P*_[*i*,*j*]_ is the elastic-optical coefficient, Δ*λ*_*B*_ is the wavelength drift value of the grating, *α* is the thermal expansion coefficient, *ζ* is the thermo-optical coefficient, and Δ*T* is the temperature change, °C.

An additional temperature sensor was used to obtain the strain change value and compensate for the influence of the temperature effect during the sensor test.

### Strain measurement of fiber Bragg grating

When the fiber grating is only subjected to stress,

$$\frac{\Delta \lambda_{B}}{\lambda_{B}}=\frac{\Delta \Lambda }{\Lambda }+\frac{\Delta n_{eff}}{n_{eff}},$$ (3)

where Δ*n*_*eff*_ is the change in the refractive index, and ΔΛ is the change in the grating period.

Assuming that the fiber grating is uniform, the relative change rate of the grating period and the relative change rate of the physical length of the grating segment are the same.

$$\frac{\Delta \Lambda }{\Lambda }=\frac{\Delta L}{L}=\varepsilon =\frac{\Delta \lambda_{B}}{\lambda_{B}(1-P_{e})},$$ (4)

where *L* is the physical length of the grating segment (mm), and Δ*L* is the change in the physical length of the grating segment (mm).

When the FBG is not under stress, the central reflection wavelength *λ*_*B*_ caused by the temperature change can be expressed as

$$\frac{\Delta \lambda_{B}}{\lambda_{B}}=(\alpha +\xi )\Delta T.$$ (5)

When stress and temperature change simultaneously, it can be expressed as

$$\frac{\Delta \lambda_{B}}{\lambda_{B}}=(1-P_{e})\varepsilon +(\alpha +\xi )\Delta T,$$ (6)

where *P*_*e*_ is the effective elasticity coefficient, and the value of the optical fiber *P*_*e*_ used in this study was 0.22 [19, 20].

The measurement formula of the fiber grating strain *ε* can be transformed into the following Formula (7) in a stable temperature environment.


$$\varepsilon =\frac{\Delta \lambda_{B}}{\lambda_{B}}\frac{1}{1-P_{e}}=1.28\frac{\Delta \lambda_{B}}{\lambda_{B}}.$$ (7)

### Analysis of strain transmission of fiber Bragg grating bolt sensor

(1) Basic assumptions of the FBG sensor

The FBG sensor constituent materials follow the following basic assumptions:

1. The fiber core is only transmitted by the uniformly strained matrix along the axis and not directly subjected to force;
2. Epoxy resin bears normal stress and shear stress, not directly subjected to stress;
3. The substrate bears a specific range of shear stress.

The three materials were all elastic, and their structures were intact, without falling off and relative slipping. Plastic deformation was not considered in this study.

**Fig 1:**
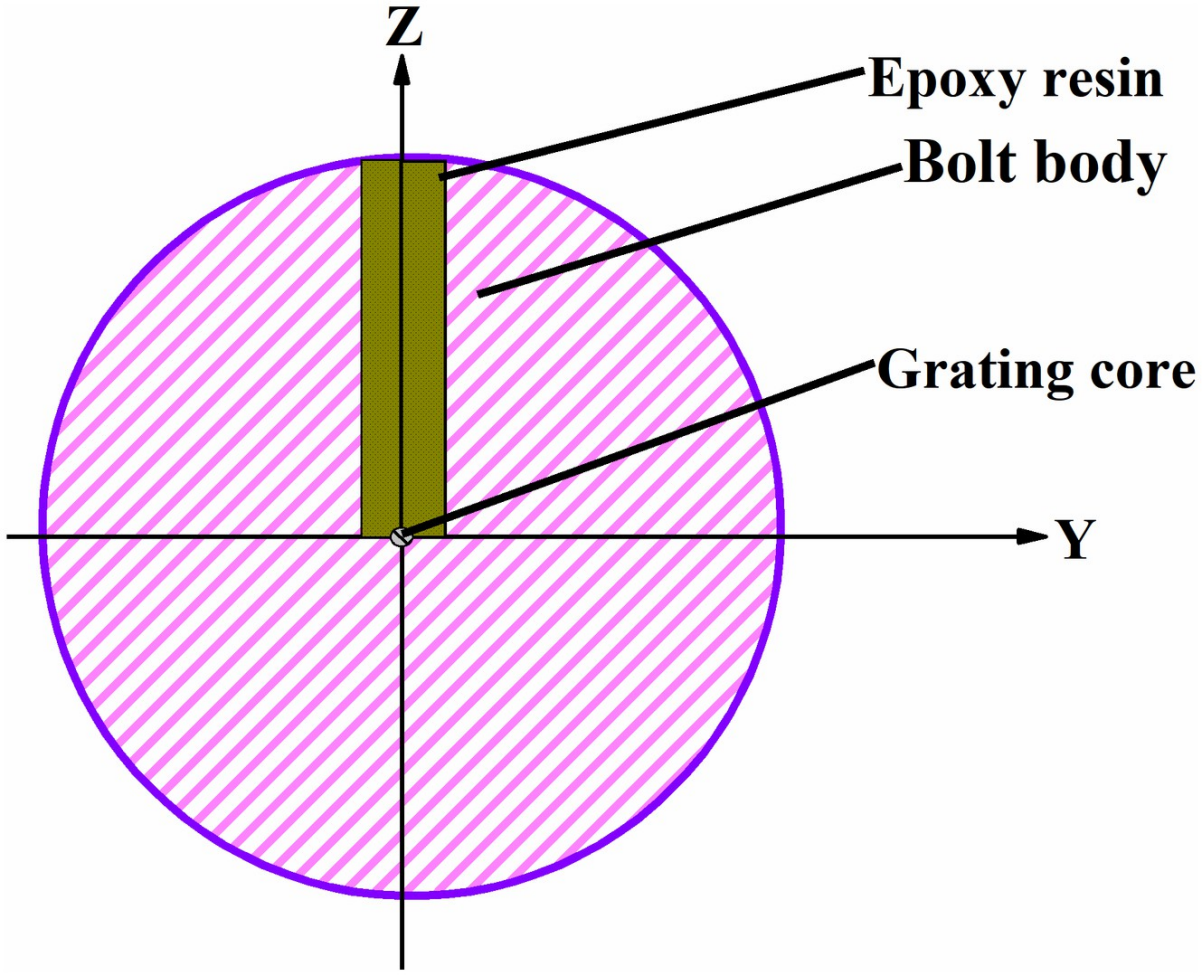
Schematic of FBG sensor embedded in the structure.

The cross section of the FBG sensor is shown in Fig 1, which comprises a bolt body, epoxy resin, and FBG.

(2) Theoretical analysis of strain transfer

**Fig 2:**
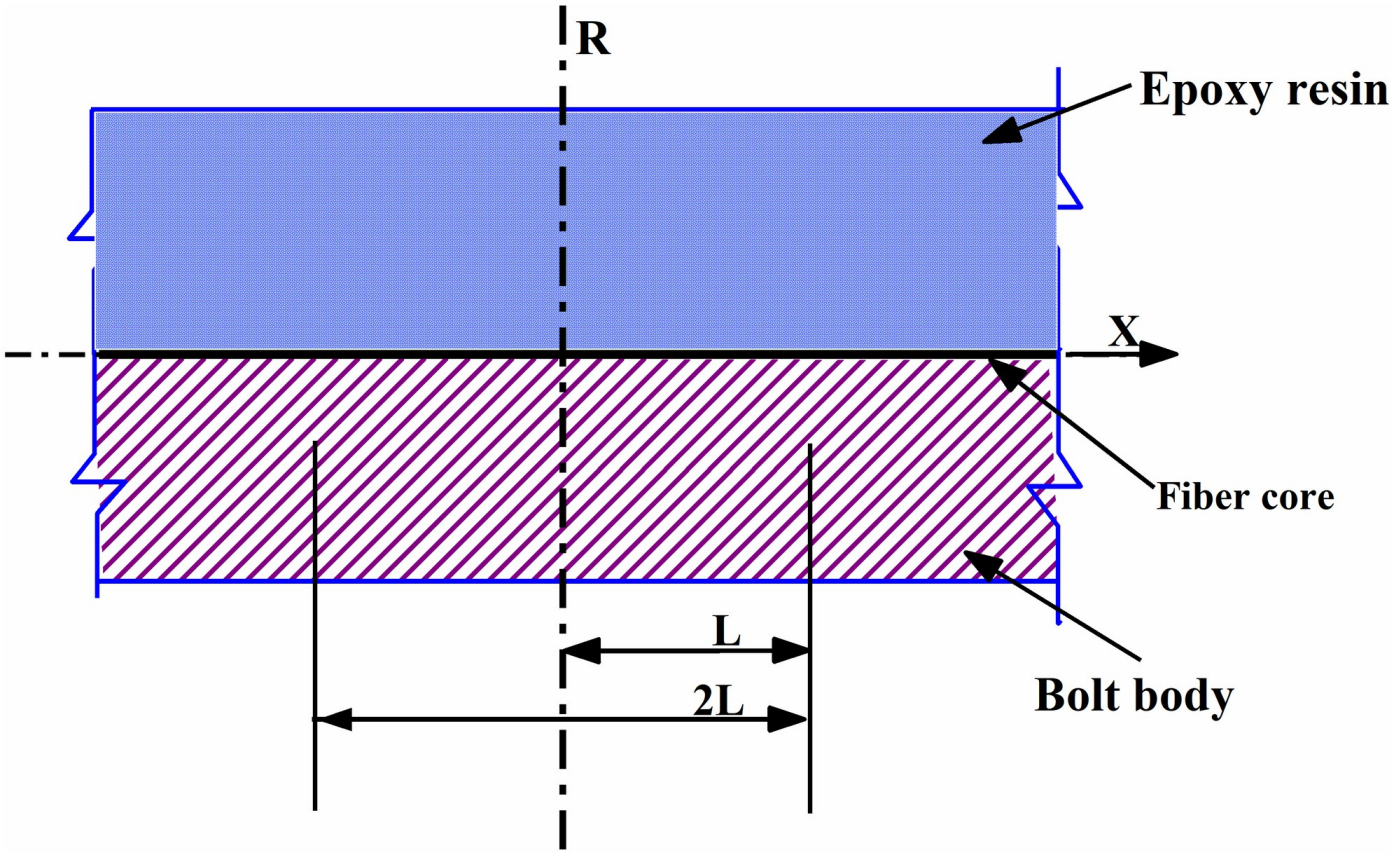
Structure profile diagram.

**Fig 3:**
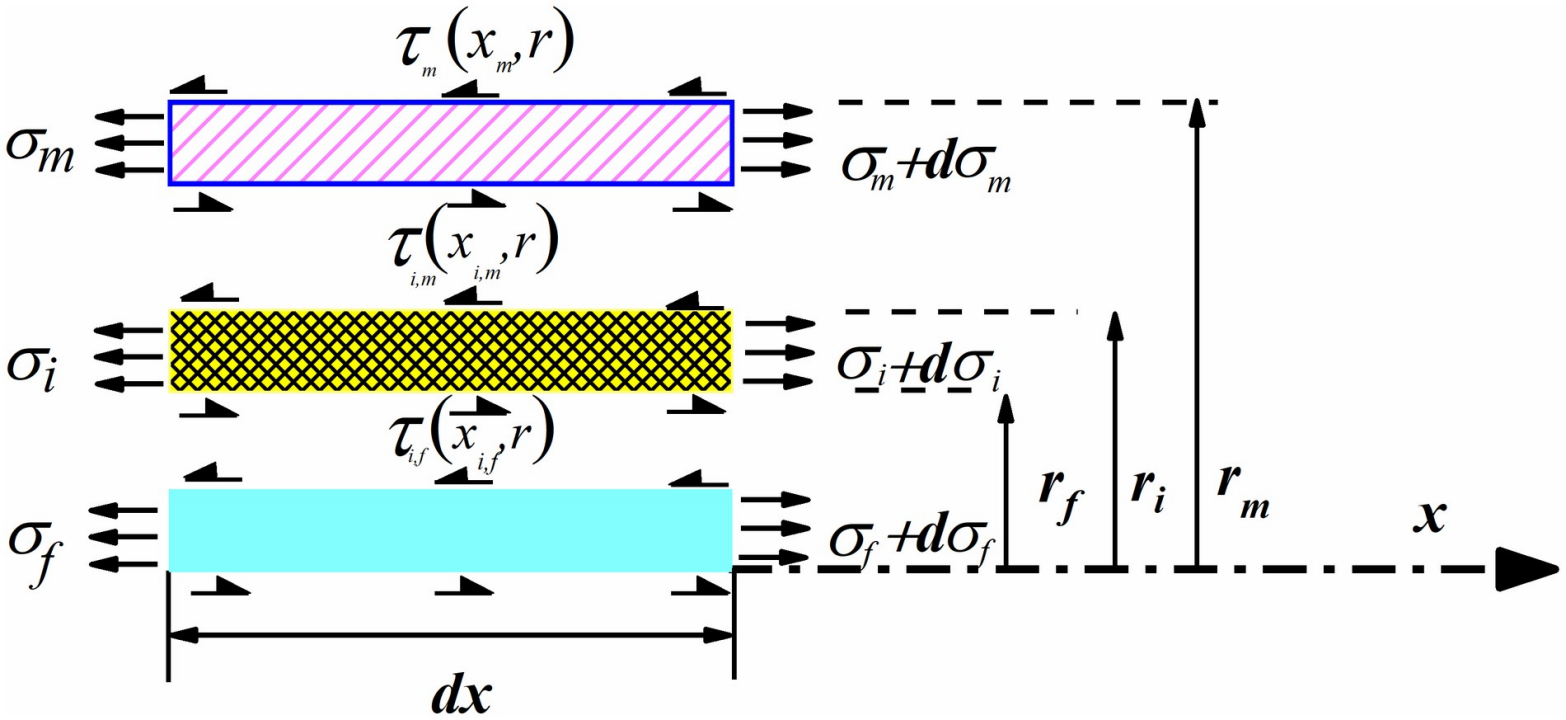
Stress distribution of fiber core, epoxy resin, and substrate.

Assuming that the gauge length of the FBG sensor area is 2 L (as shown in Fig 2), the outer side of the matrix structure *r*_*m*_ bears uniform axial stress, and the force of the three layers of materials is shown in Fig 3. The direction *X* represents the axis direction of the FBG sensor.

For the fiber core microsegment, the force balance equation in the *X* direction is [21]:

$$\tau_{i,f}(x,r_{f})=\frac{1}{2dx}r_{f}d\sigma_{f}.$$ (8)

For the epoxy micro-segment [21], the radius is from *r*_*f*_ to *r* (*r*_*f*_ < *r* < *r*_*i*_), the resultant force along the direction *X* is zero, and we have:

$$\tau_{i}(x,r)=\frac{r_{f}}{r}\tau_{i,f}(x,r_{f})+\frac{r^{2}-r_{f}{}^{2}}{2r}\frac{d\sigma_{i}}{dx},$$ (9)

where *r*_*f*_ is the radius of the fiber core and *r*_*i*_ is the radius of the middle layer.

For micro-segment of the matrix structure, we have:

$$\tau_{m}(x,r)=E_{f}\frac{r_{i}}{r}(1-\frac{r^{2}-r_{i}{}^{2}}{r_{m}{}^{2}-r_{i}{}^{2}})(\frac{r_{f}}{r_{i}}\frac{r_{f}}{2dx}d\varepsilon_{f}+\frac{r_{i}{}^{2}-r_{f}{}^{2}}{2r_{i}}\frac{E_{i}}{E_{f}}\frac{d\varepsilon_{i}}{dx}),$$ (10)

where *E*_*f*_ is the core elastic modulus (GPa), *E*_*i*_ is the epoxy elastic modulus (GPa), *ε*_*f*_ is the axial strain of the fiber core (10^−6^), *ε*_*i*_ is the axial strain of the middle layer (10^−6^), *σ* is the normal stress, *τ* is the shear stress, *r* is the radius of each layer, *m* is the matrix-related variable, *i* is the epoxy-related variable, and *f* is the core-related variable.

Set the following parameters.


$$k^{-2}=\frac{1}{2}r_{f}{}^{2}E_{f}\{\frac{1}{G_{i}}\text{ln}(r_{i}/r_{f})+\frac{1}{G_{m}}[\frac{r_{m}{}^{2}}{r_{m}{}^{2}-r_{i}{}^{2}}\text{ln}(r_{m}/r_{i})-\frac{1}{2}]\}.$$ (11)

It can be deduced that the axial strain of the core is

$$\varepsilon_{f}=\varepsilon_{m}(1-\frac{cosh(kx)}{cosh(kL)}).$$ (12)

The material mechanical property parameters of the epoxy resin, plexiglass, and bolt body are listed in Table 1.

**Table 1:** Mechanical properties of the core, epoxy resin, and the base structure.

Material parameters		Symbol	Numerical value	Unit
Young’s modulus of elasticity	Fiber core	*E*_*f*_	73	GPa
	Epoxy resin	*E*_*i*_	4	GPa
	Axle steel	*E*_*mr*_	206	GPa
	FRP	*E*_*mg*_	72	GPa
The radius of each layer	Fiber core	*r*_*f*_	4.5×10^−3^	mm
	Epoxy resin	* $r_{i}^{+y}$ *	1	mm
	Axle steel	* $r_{i}^{+z}$ *	9	mm
	FRP	*r*_*m*_	9	mm
	Fiber core	*r*_*m*_	16	mm
Shear modulus of elasticity	Fiber core	*G*_*f*_	31.2	GPa
	Epoxy resin	*G*_*i*_	1.35	GPa
	Axle steel	*G*_*m*_	79	GPa
	FRP	*G*_*i*_	30	GPa
Poisson’s ratio	Fiber core	*μ*_*f*_	0.30	—
	Epoxy resin	*μ*_*i*_	0.48	—
	Axle steel	*μ*_*mr*_	0.17	—
	FRP	*μ*_*mg*_	0.2	—

**Fig 4:**
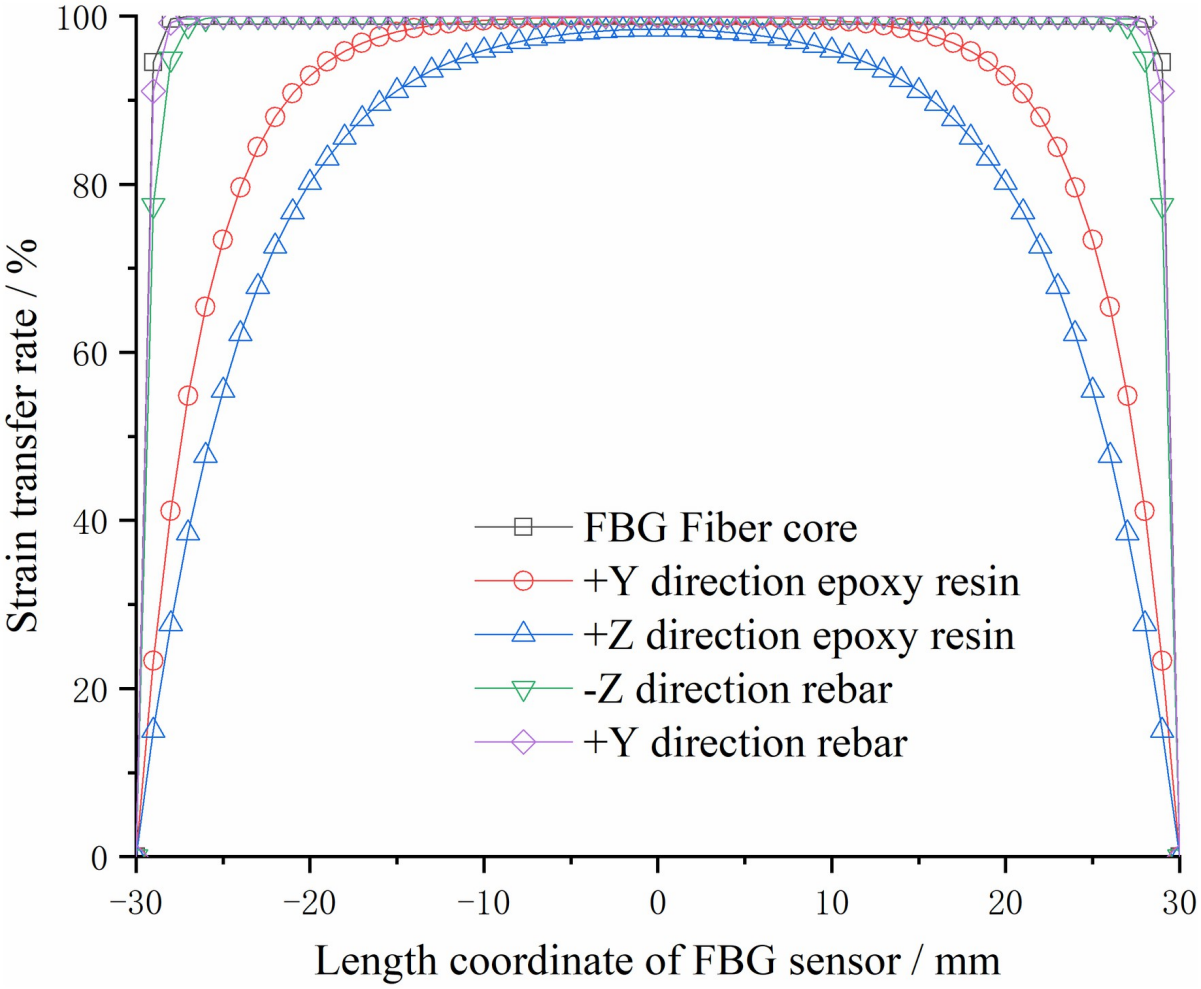
Relationship curve between strain transfer rate of steel substrate and length coordinate.

**Fig 5:**
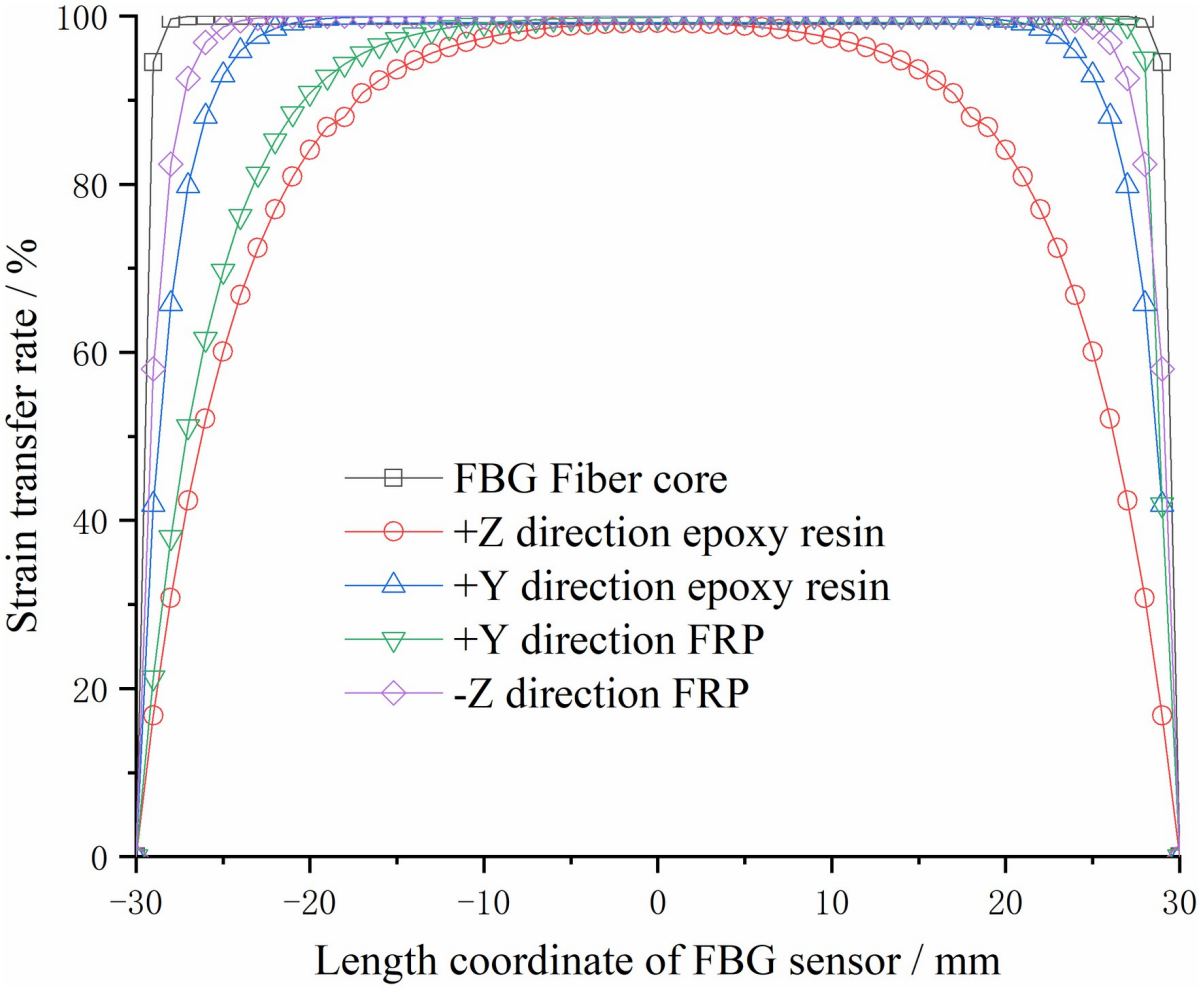
Relationship curve between strain transfer rate of FRP substrate and length coordinate.

The different shear elastic moduli of the two matrix structures are the same in all directions, and the strain transfer rate at each point of the FBG sensor within the gauge length is shown in Figs 4 and 5.

As shown in Figs 4 and 5, the abscissa is the distance from the center of the FBG sensor, and the ordinate is the strain transfer rate of different materials in different directions. From the analysis of the strain transfer rate of the two different materials, it can be observed that the farther the FBG sensor, the smaller the strain transfer rate; the closer to the FBG sensor, the greater the strain transfer rate, and the two ends are zero. The largest strain transfer rate occurred at the center point. The changing trend close to the center point is slower than that at the farther position of the sensor center point. It shows that within the distance of the gate area (the length of the gate area is 10 mm, i.e., the abscissa of the figure is -5–5 mm), the strain transmission loss between each material is minimal. The stress acting on the bolt can be accurately reflected by the wavelength change of the FBG sensor.

### Designation of fiber Bragg grating bolt

**Fig 6:**
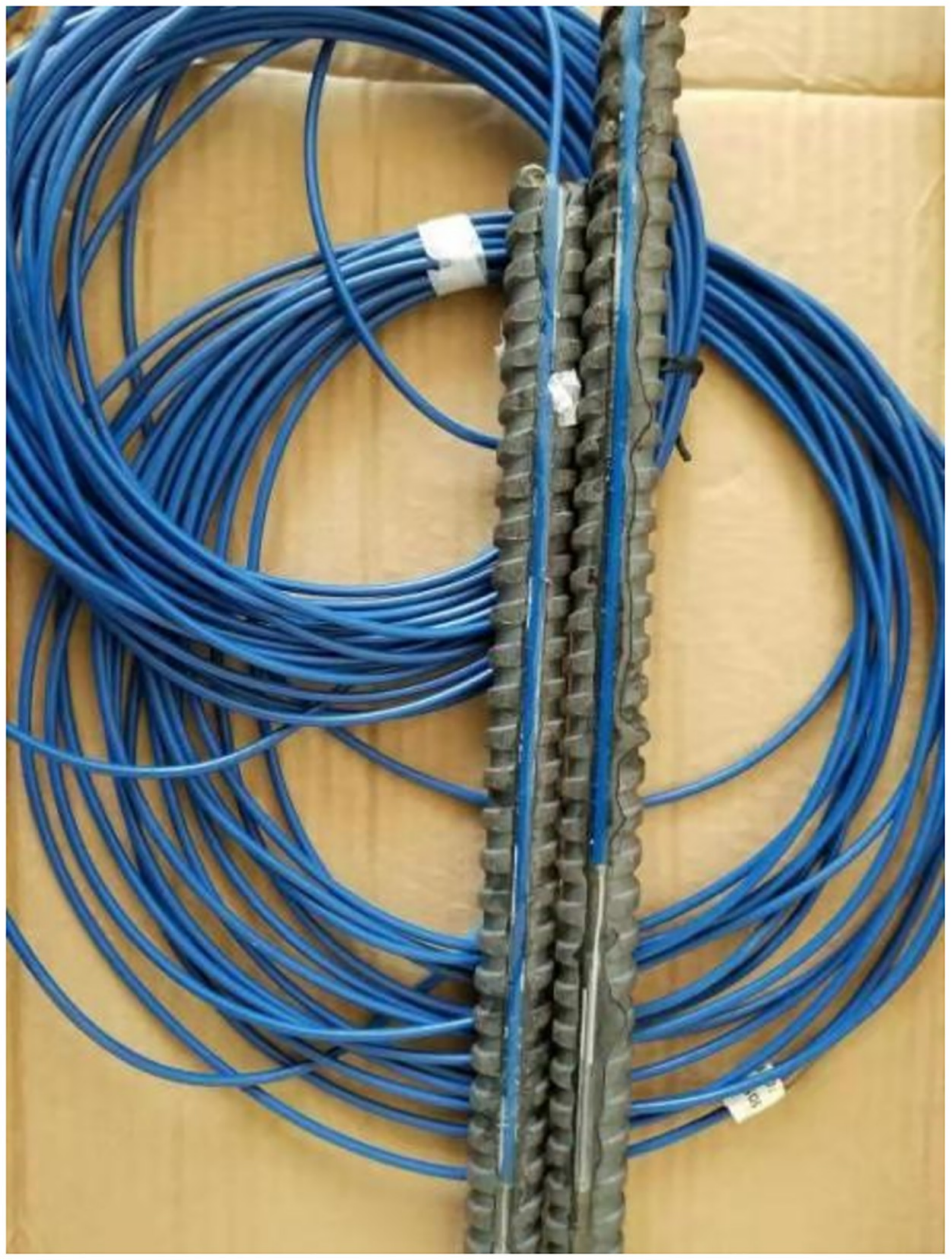
The bolt with embedded sensor.

To ensure that the sensor is not damaged when the bolt is installed and can effectively monitor the force and deformation of the bolt support structure of the roadway, it is embedded in the bolt and encapsulated, as shown in Fig 6. It was then installed in the coal and rock together with the bolt.

**Fig 7:**
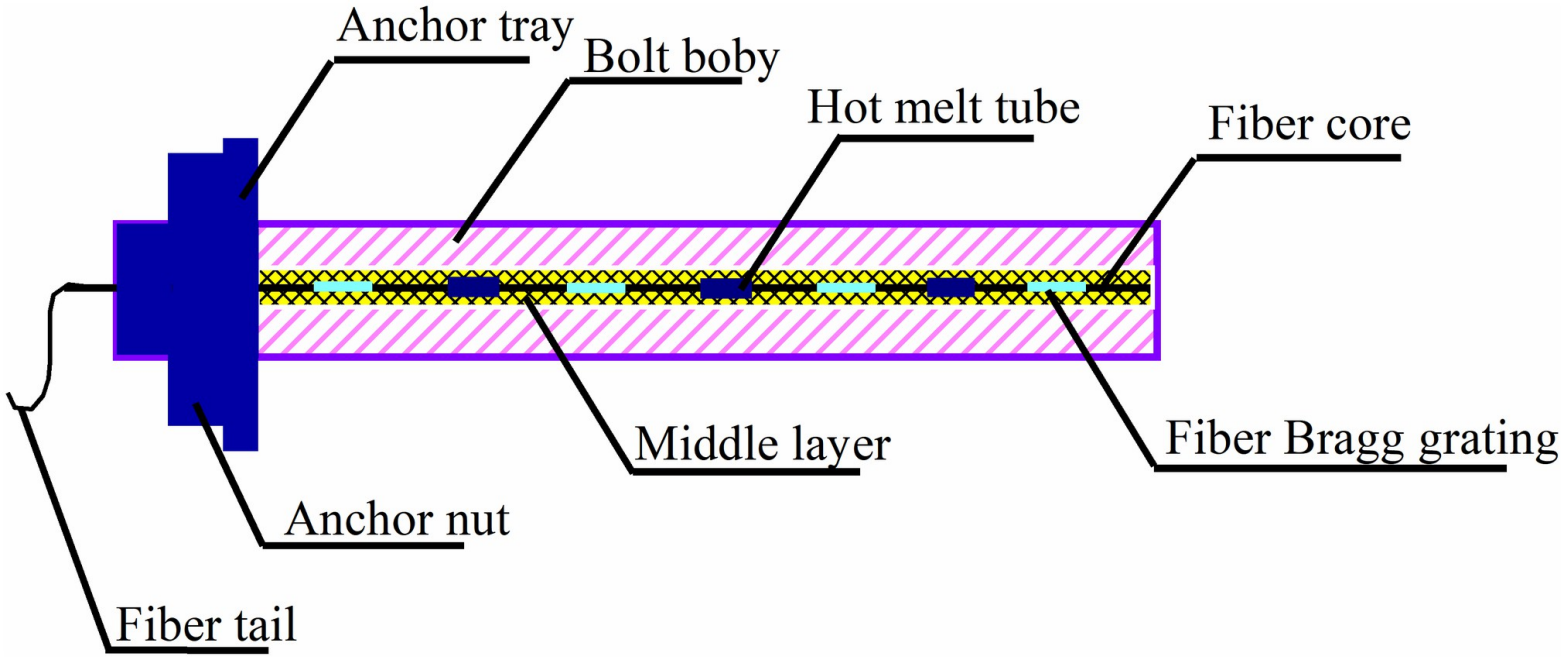
Fiber grating sensor structure layout diagram.

A minor groove with a width of 2 mm and a depth of the radius of the bolt was machined on an 18 mm diameter screw steel bolt and a 32 mm diameter FRP bolt. If it is a screw steel bolt, the depth is 9mm; if it is a FRP bolt, the depth is 16mm. The FBG sensor is tightly pasted on the bottom of the groove, arranged and packaged in the bolt, and the armored optical cable was drawn out at the tail. The FBG modem was connected for real-time monitoring, as shown in Fig 7.

**Fig 8:**
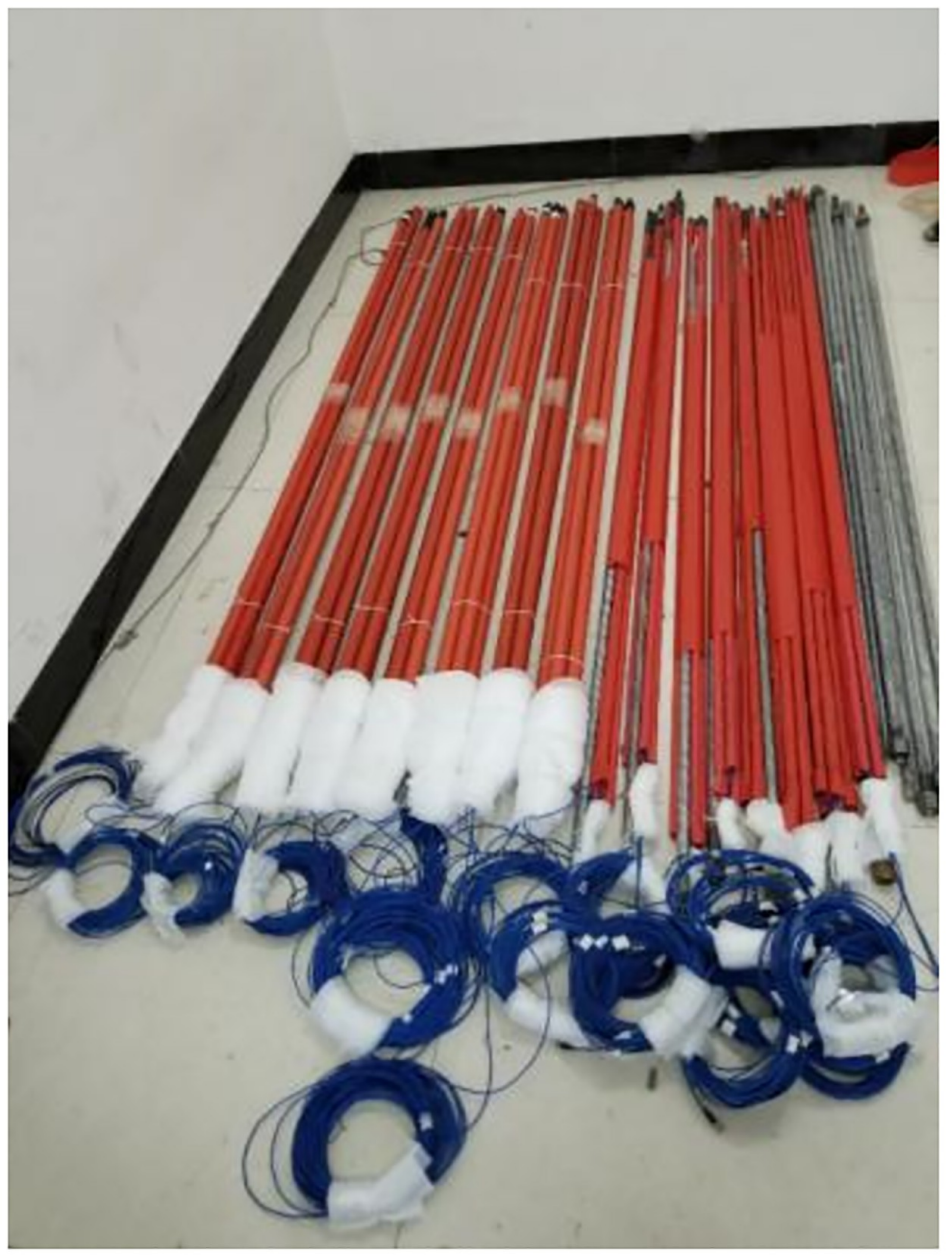
Protection groove of sensor jumper head.

To prevent accidents during the transportation of the sensor, a thermoplastic tube was used for sealing. The bolt connection position and the tail fiber were wrapped with plastic foam to prevent damage to the sensor during transportation or downhole transportation, as shown in Fig 8.

### Incremental elastic modulus of the bolt

From Eq (7), the axial force of the bolt can be expressed as

$$F=AE\varepsilon =1.28EA\frac{\Delta \lambda_{B}}{\lambda_{B}},$$ (13)

where *F* is the axial force of the bolt (N), *E* is the elastic modulus of the bolt (MPa), and *A* is the cross-sectional area of the bolt (m^2^).

When the axial force value of the bolt is small, the change in its cross-sectional area is also small, which can be ignored. Therefore, *A* can be considered constant. After the bolt material was determined, its elastic modulus remained unchanged.

**Fig 9:**
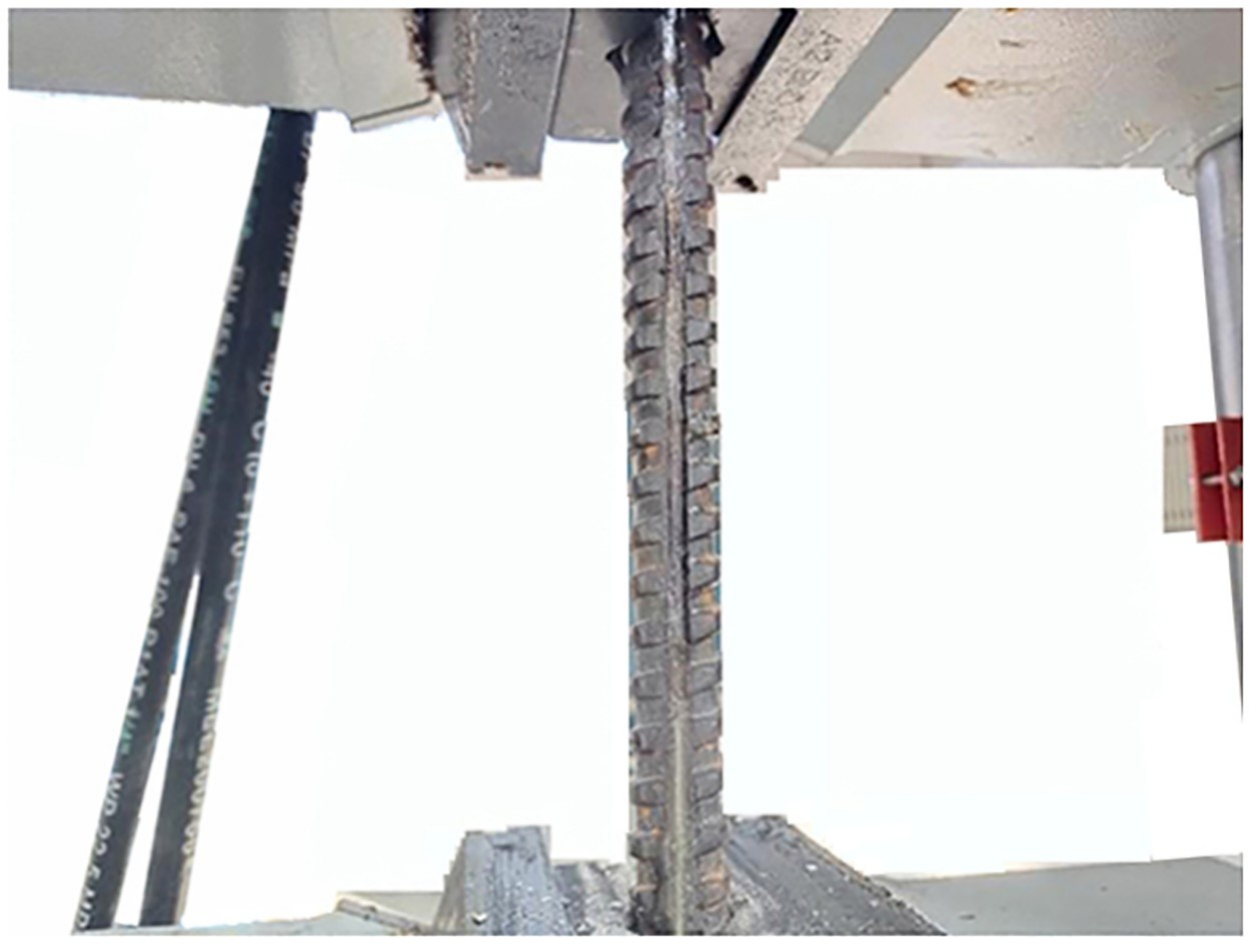
Steel bolt tensioned on the test machine.

**Fig 10:**
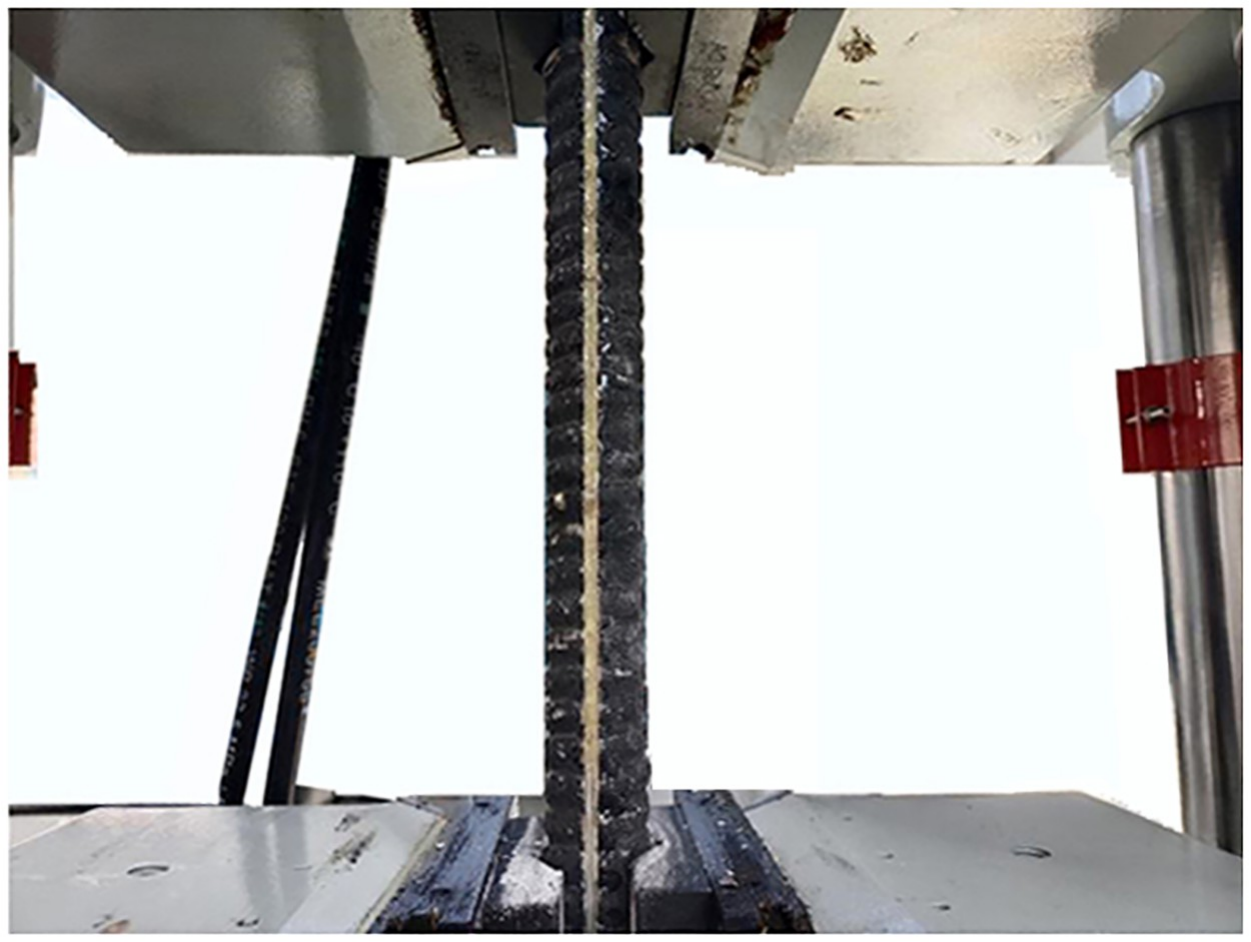
FRP bolt tensioned on the test machine.

**Fig 11:**
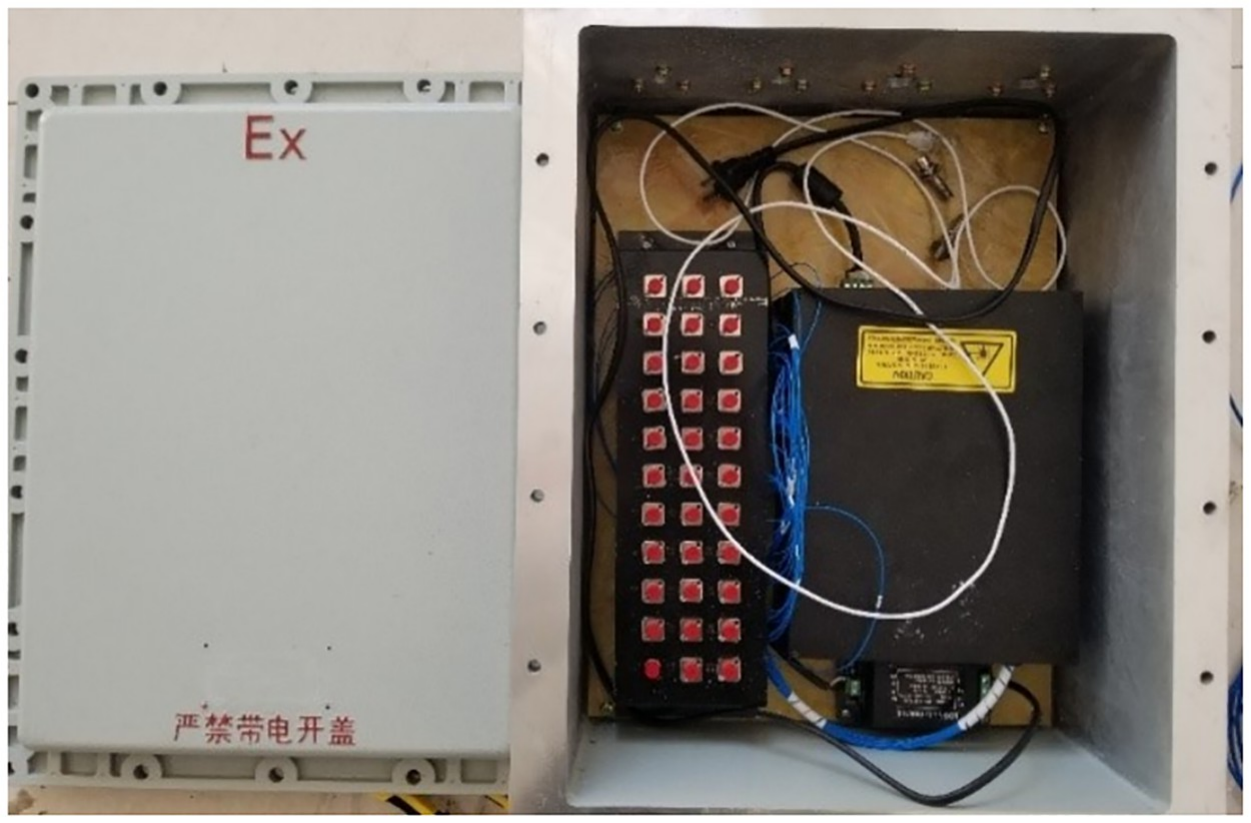
The demodulator.

**Fig 12:**
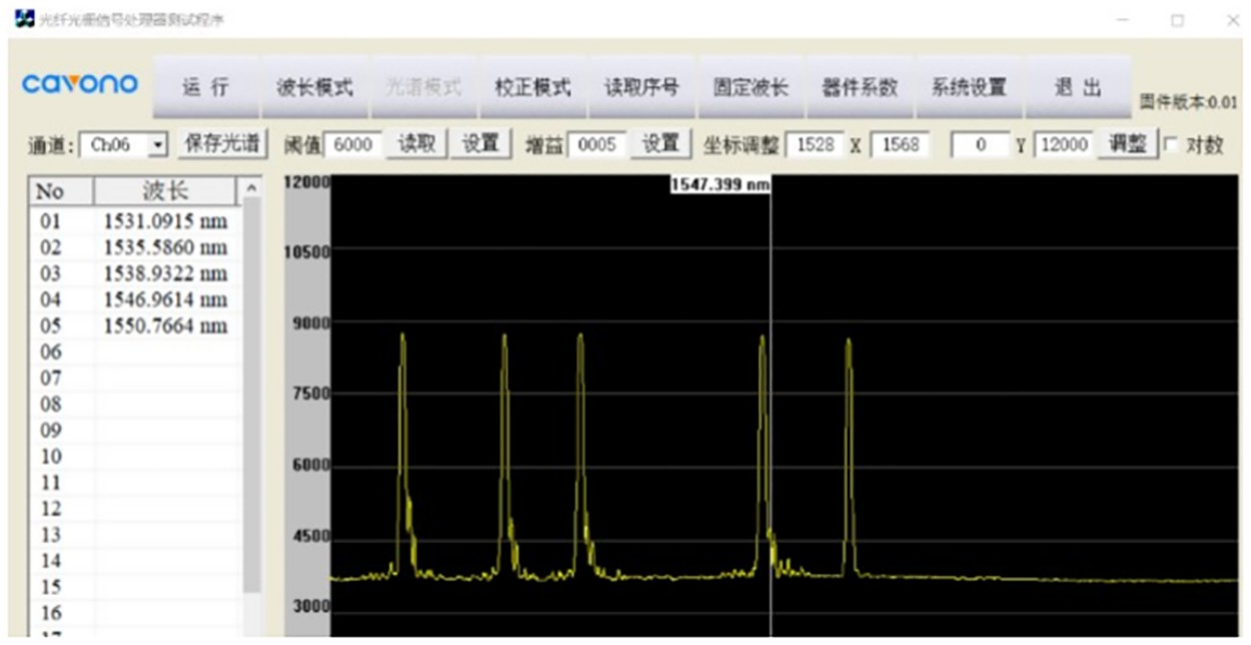
The monitoring data of central wavelength.

**Fig 13:**
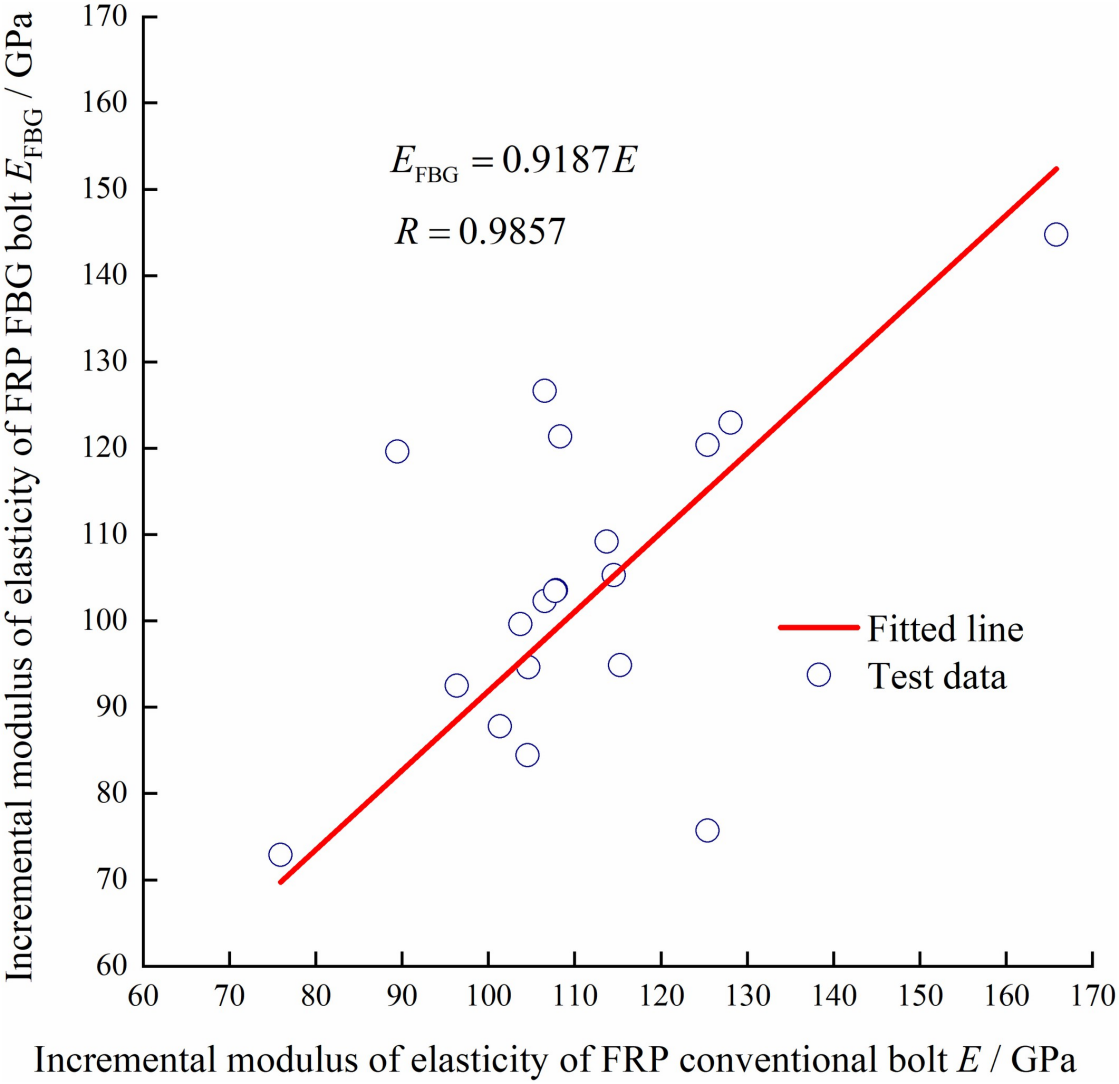
FRP conventional bolt incremental elastic modulus relation curve.

**Fig 14:**
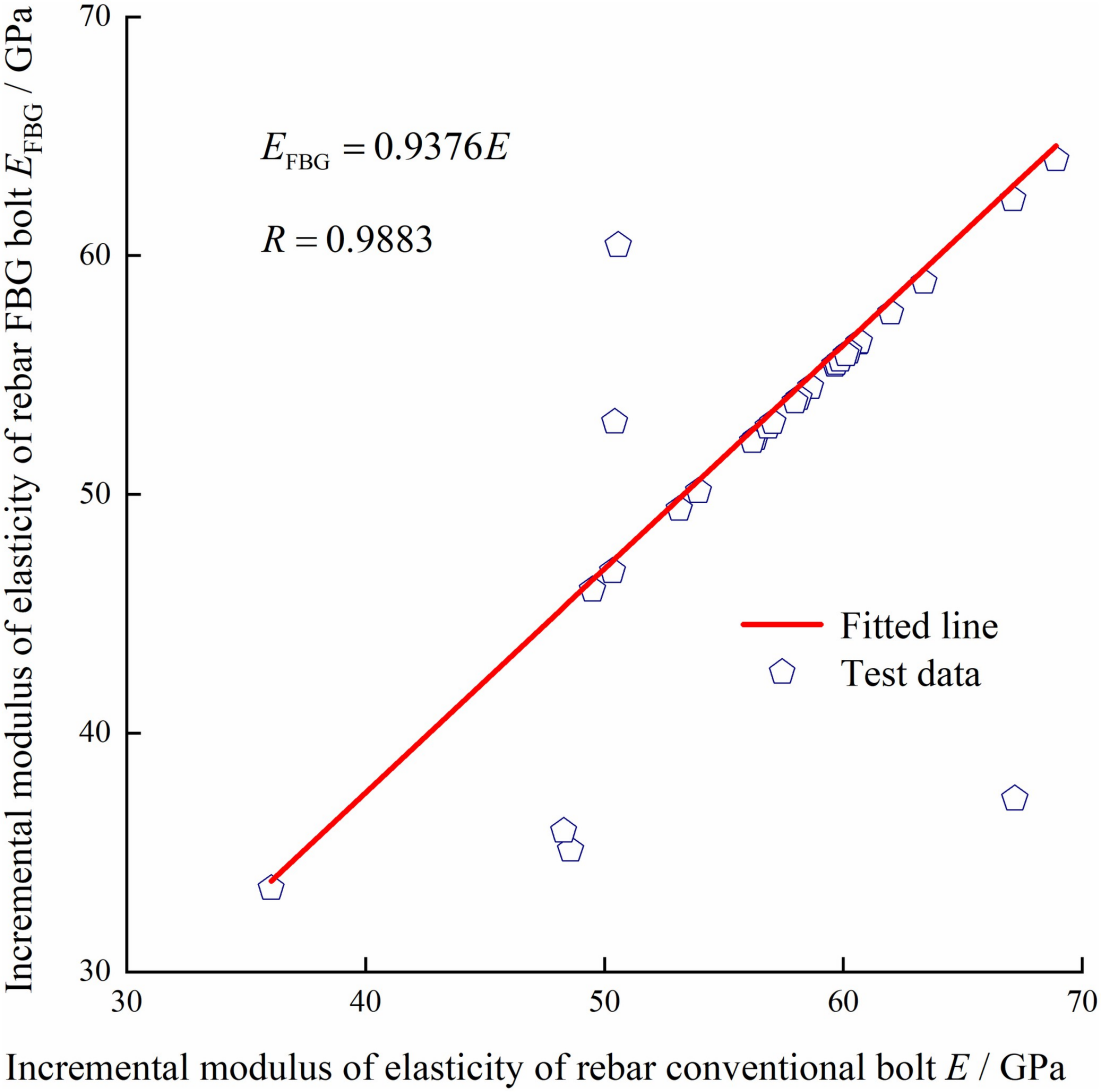
Rebar conventional bolt incremental elastic modulus relation curve.

With constant temperature and constant speed loading, two types of conventional bolts and two types of FBG bolts are stretched on the electro-hydraulic servo universal testing machine, as shown in Figs 9–12. According to the measured data of the center wavelength of the FBG sensor, the relationship curves between the conventional and incremental elastic moduli of the FBG bolt are drawn, as shown in Figs 13 and 14.

In the elastic deformation stage, after the bolt is stretched and before yielding, the ratio of the incremental value of the axial force change to the incremental value of strain is the incremental elastic modulus of the bolt body. From the distribution of the test data in Figs 13 and 14, it can be observed that the incremental elastic moduli of the conventional rebar bolt and the FBG bolt are consistent, which is consistent with the material mechanical properties that the elastic modulus is the same in the elastic deformation stage. The calculated average values of the incremental elastic modulus of the conventional rebar bolt and FBG bolt are 54.2 GPa and 50.4 GPa, respectively. In addition, the average value of the incremental elastic modulus of the FRP conventional bolt and FBG bolt are 112.2 GPa and 107.7 GPa, respectively. The strength of FBG bolts can meet the actual requirements of the roadway. The test results verified the reliability of the FBG bolt sensor for monitoring the axial force state of the bolt.

### Monitoring sensor installation

**Fig 15:**
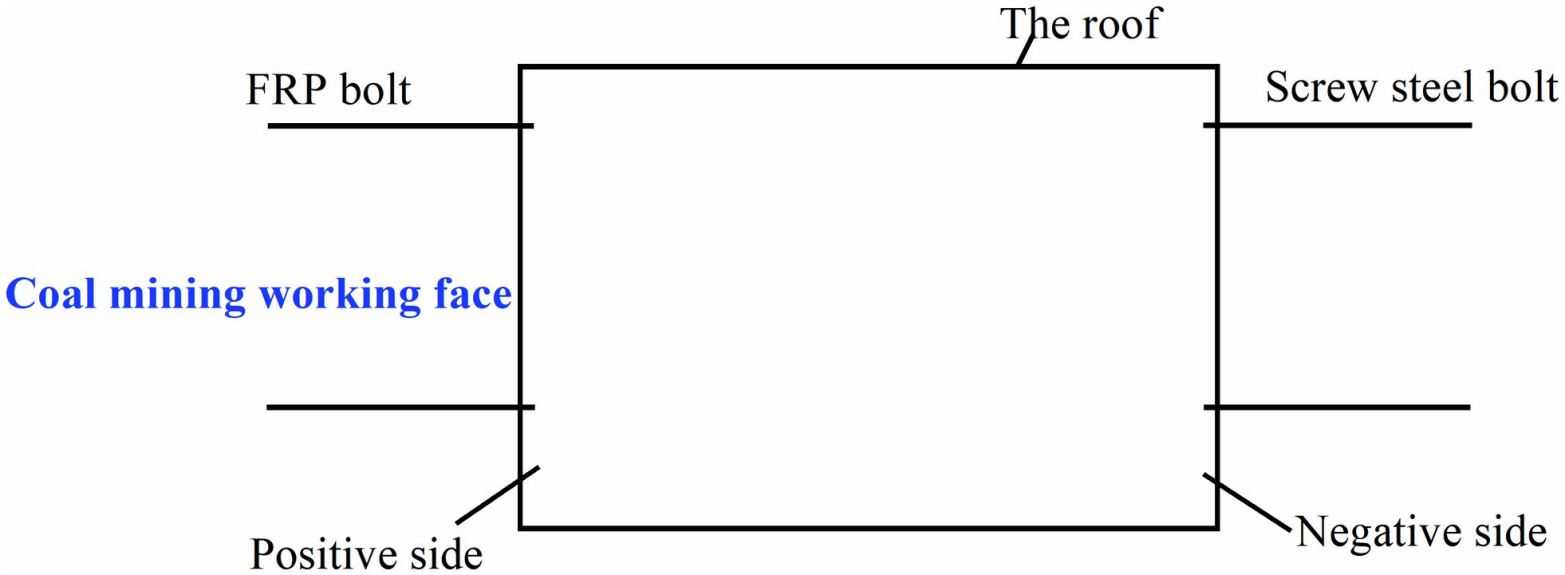
The positive and negative sides.

The bolt damage caused by the periodic pressure of the roof is mainly concentrated on the positive and negative sides of the roadway along with transportation and auxiliary transportation. The positive and negative sides are shown in Fig 15.

**Fig 16:**
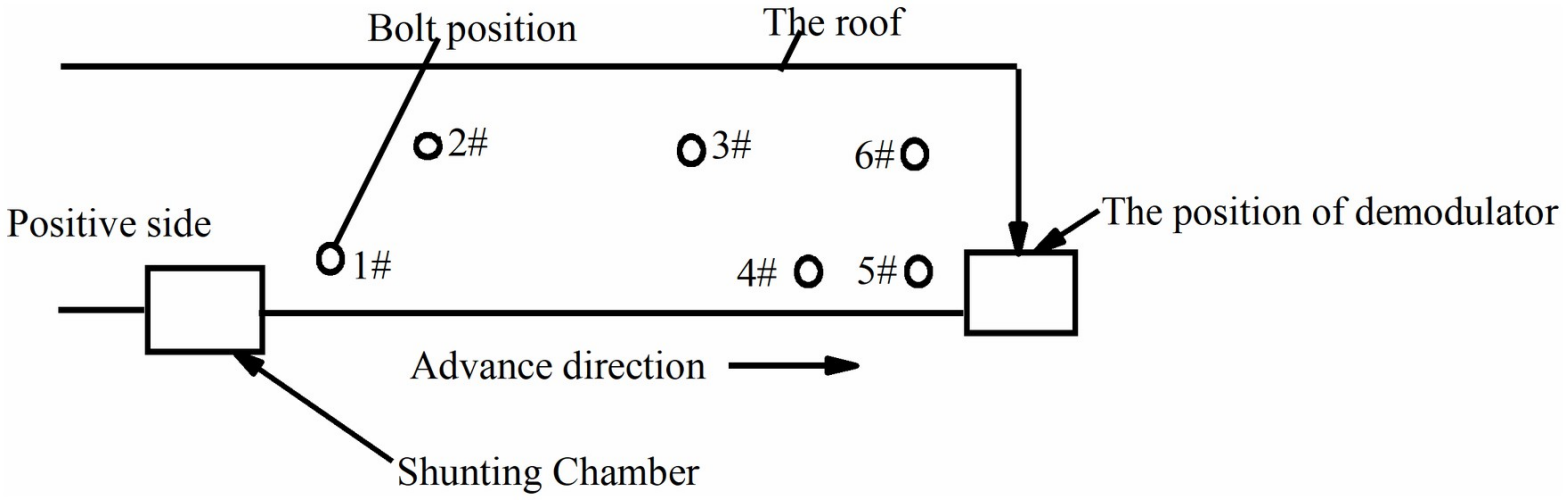
Position of bolt installation of positive side.

**Fig 17:**
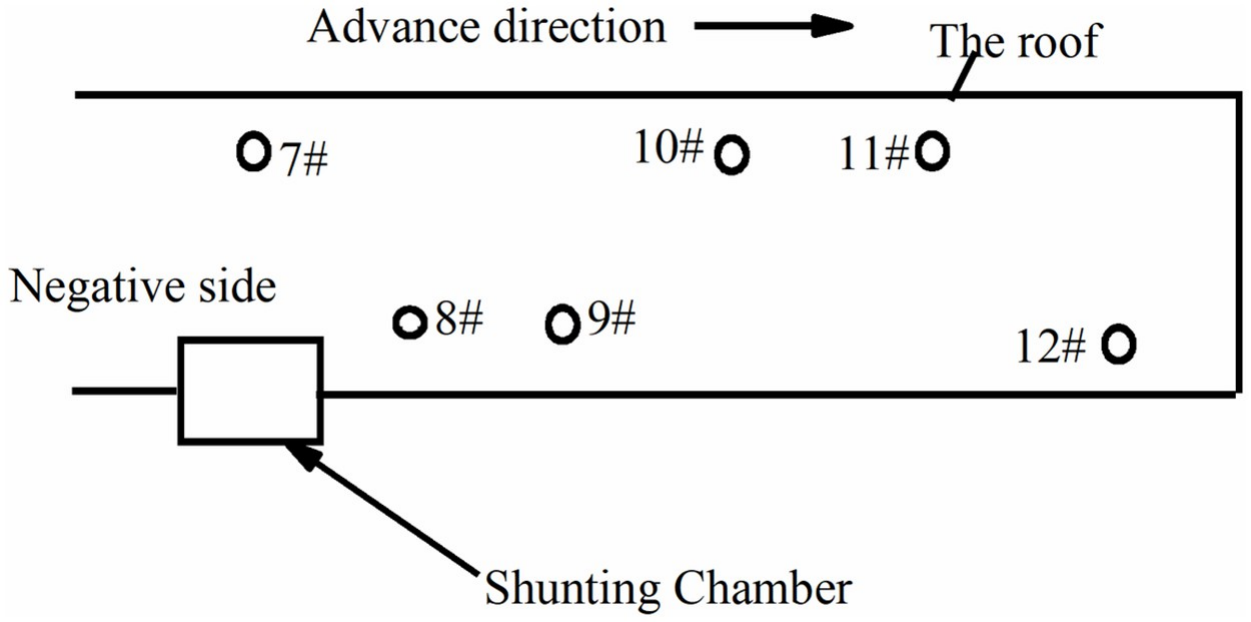
Position of bolt installation of negative side.

The maximum step distance of the periodic pressure is 15 m. Within the range of the monitoring cycle, there is a law between the deformation of the roadway support bolt and the rock pressure during the advancement of the working face, that is, the FBG sensor bolt must be placed approximately within 225 m in front of the working face. The screw steel bolts and FRP bolts were driven in sequence on the positive and negative sides of the roadway, as shown in Figs 16 and 17.

To consider the interference of roadway facilities on the positive and negative sides, the position of the fiber grating bolts was adjusted according to the scene. The driving positions of the positive and negative sides of the bolt are shown in Figs 16 and 17.

**Fig 18:**
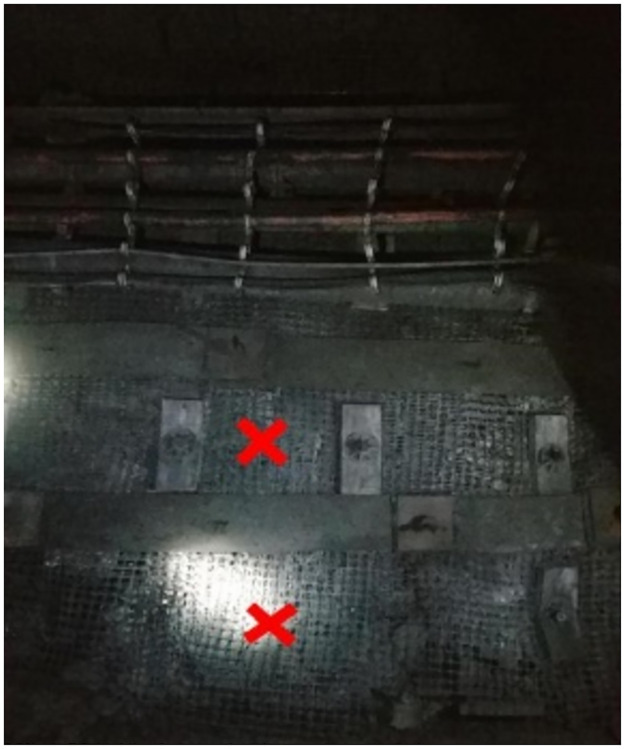
Bolt arrangement position of positive side.

**Fig 19:**
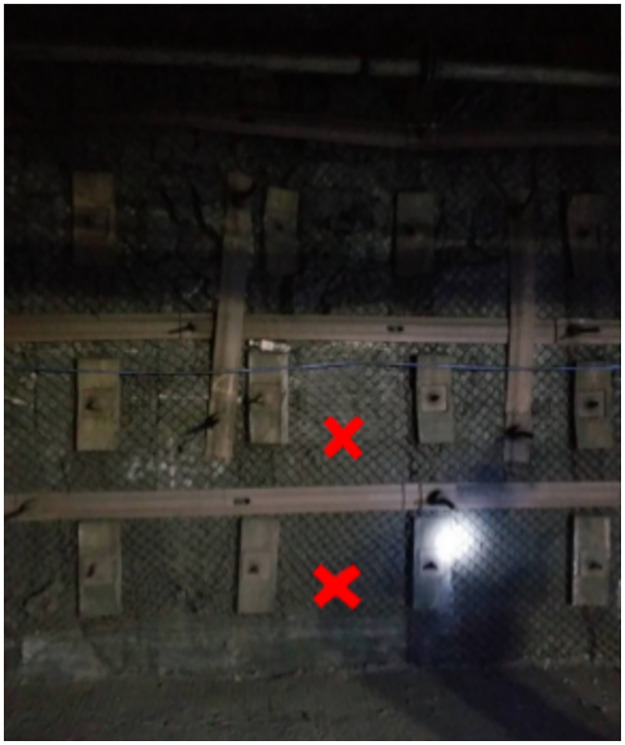
Bolt arrangement position of negative side.

Monitoring of surrounding rock roadways is a long and slow process. To make the monitoring data more accurate, it is necessary to descend the well simultaneously every day, record the real-time conditions of the underground, ensure the consistency of the external environment, and obtain real-time and accurate bolt data. The bolt arrangement position of positive and negative side is shown in Figs 18 and 19.

**Fig 20:**
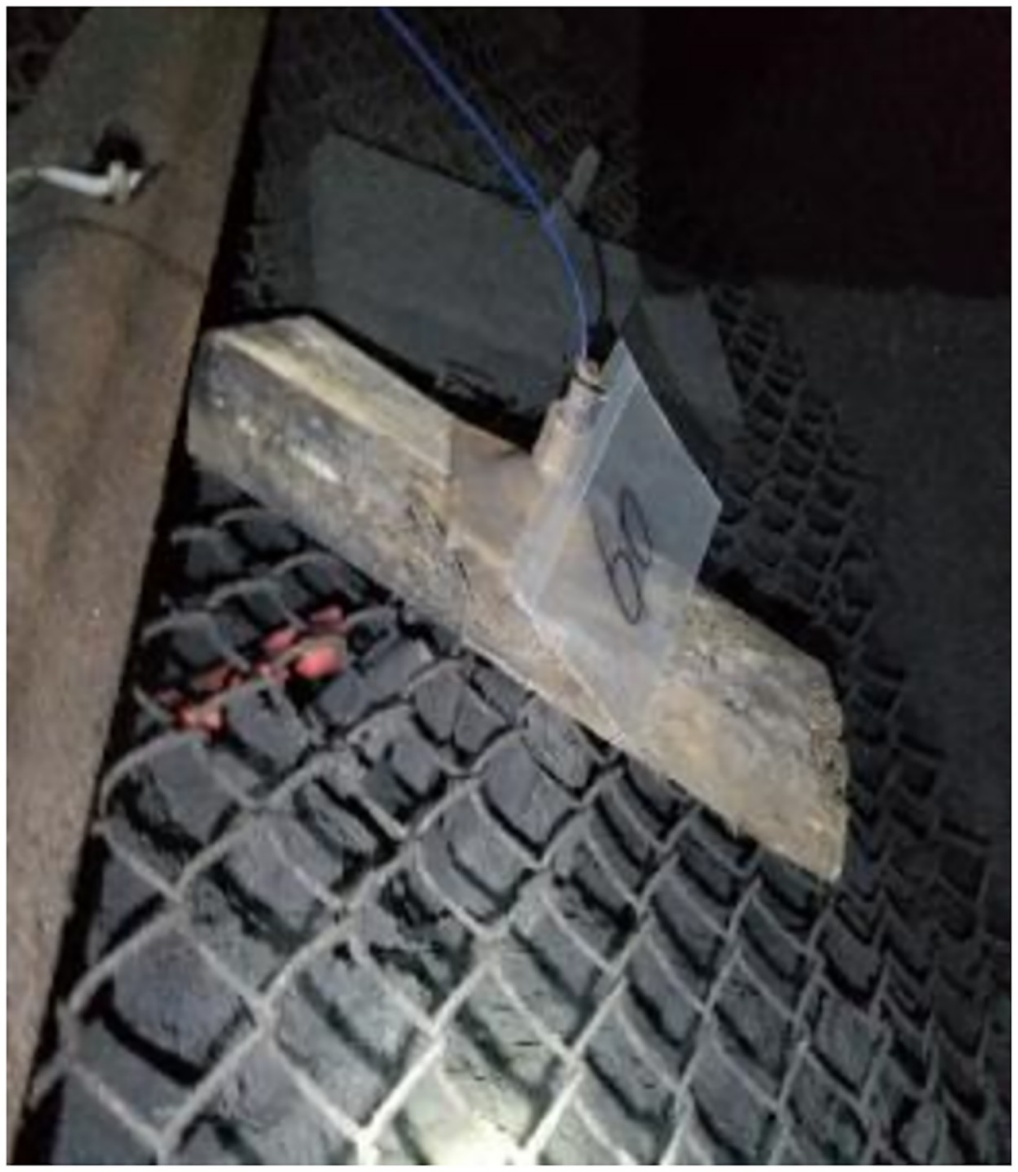
One of the bolt installation diagrams.

**Fig 21:**
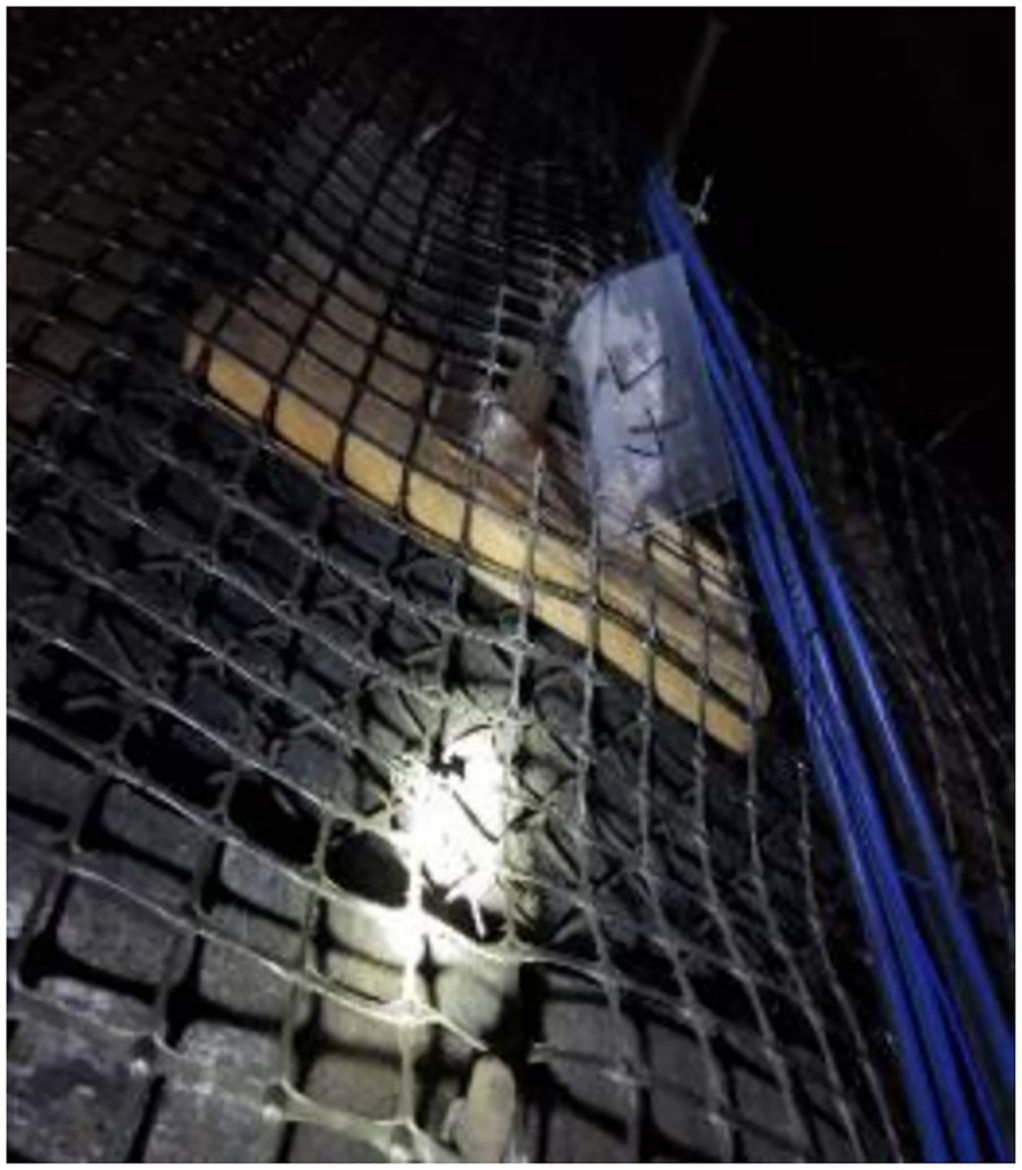
Another of the bolt installation diagrams.

This is because the bolt was driven by the manual hand-drilling method. After drilling, the anchoring resin was stirred with a stirring bolt. Thereafter, the fiber grating bolt was driven. The installation of the bolt is shown in Figs 20 and 21.

**Fig 22:**
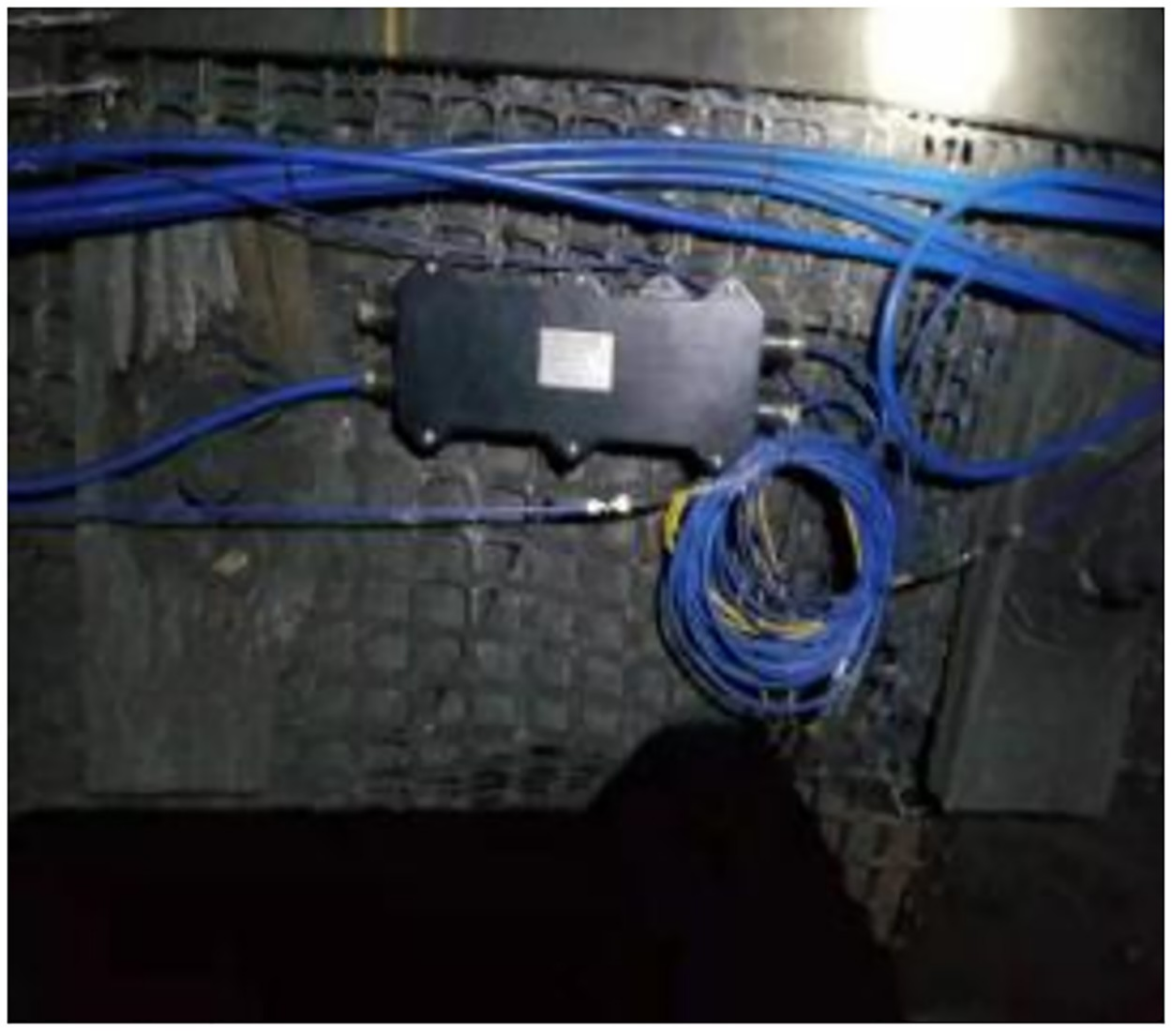
One of pictures of monitoring of connection lines.

**Fig 23:**
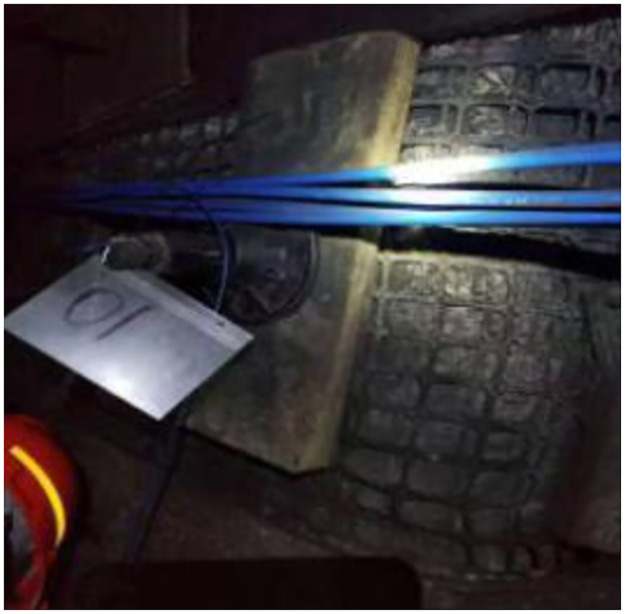
Another of pictures of monitoring of connection lines.

After the bolts were driven into the roadway, each side of the side bolts was connected to MGTS-12B mining single-mode optical cables. Simultaneously, the mining optical cables are routed near the demodulator and converged in the junction box, as shown in Figs 22 and 23.

## Force and deformation characteristics of the bolt body

### Analysis of monitoring results

Because the temperature of the underground roadway was relatively constant, and after the bolt was buried in the surrounding rock, the temperature was stable, the influence of the temperature on the sensor was negligible.

**Fig 24:**
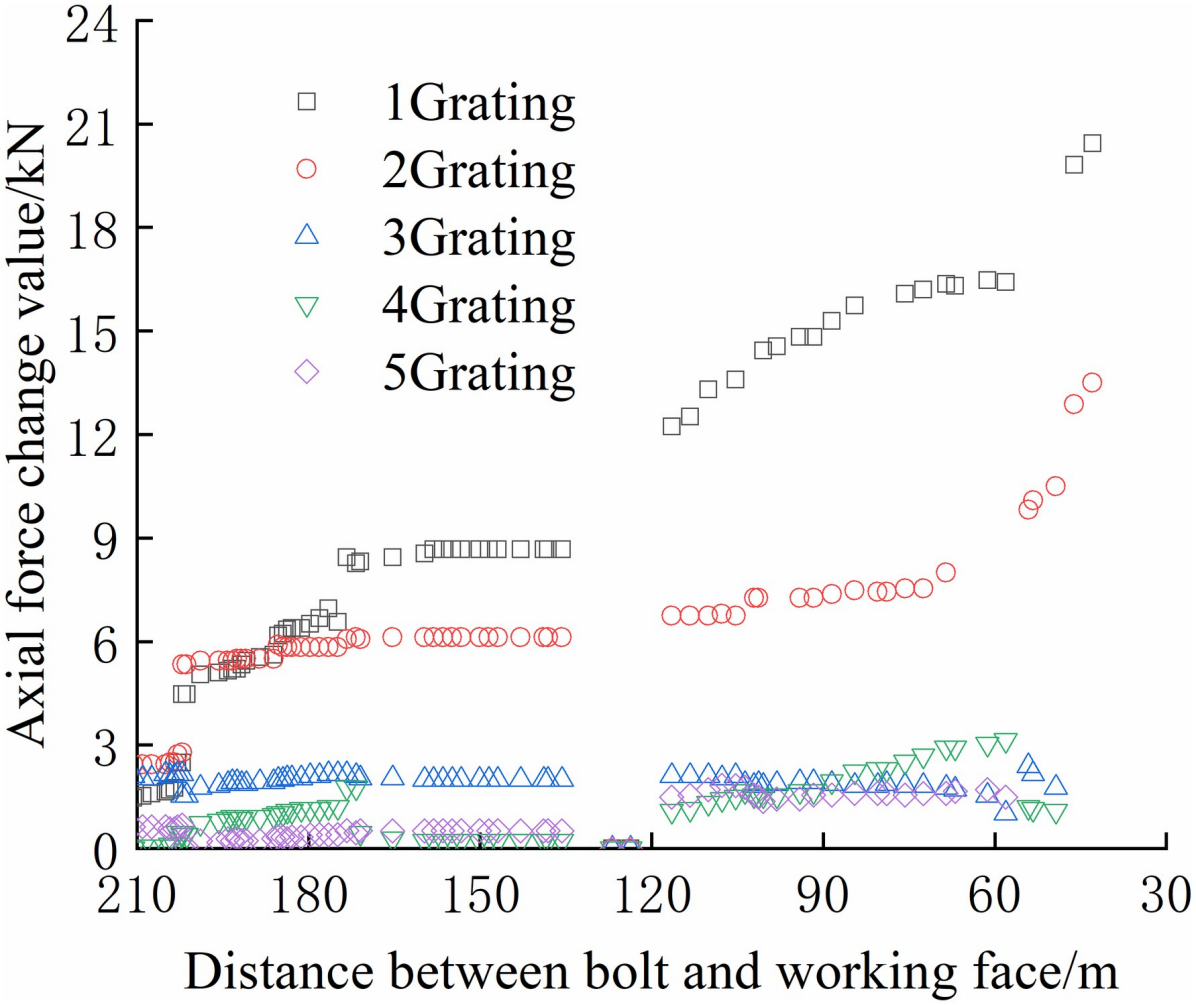
Axial stress monitoring test curve of No.1 bolt.

**Fig 25:**
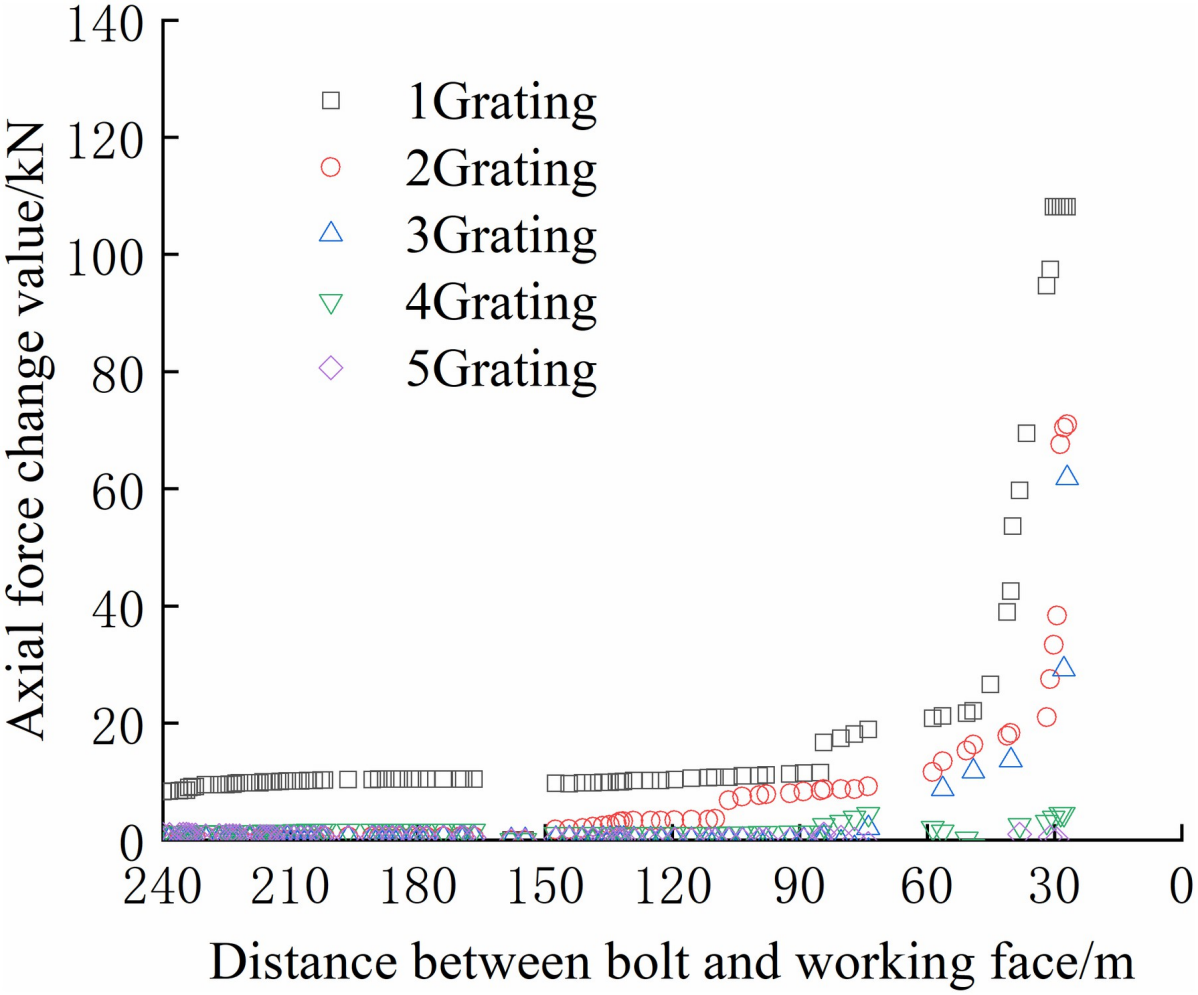
Axial stress monitoring test curve of No.2 bolt.

**Fig 26:**
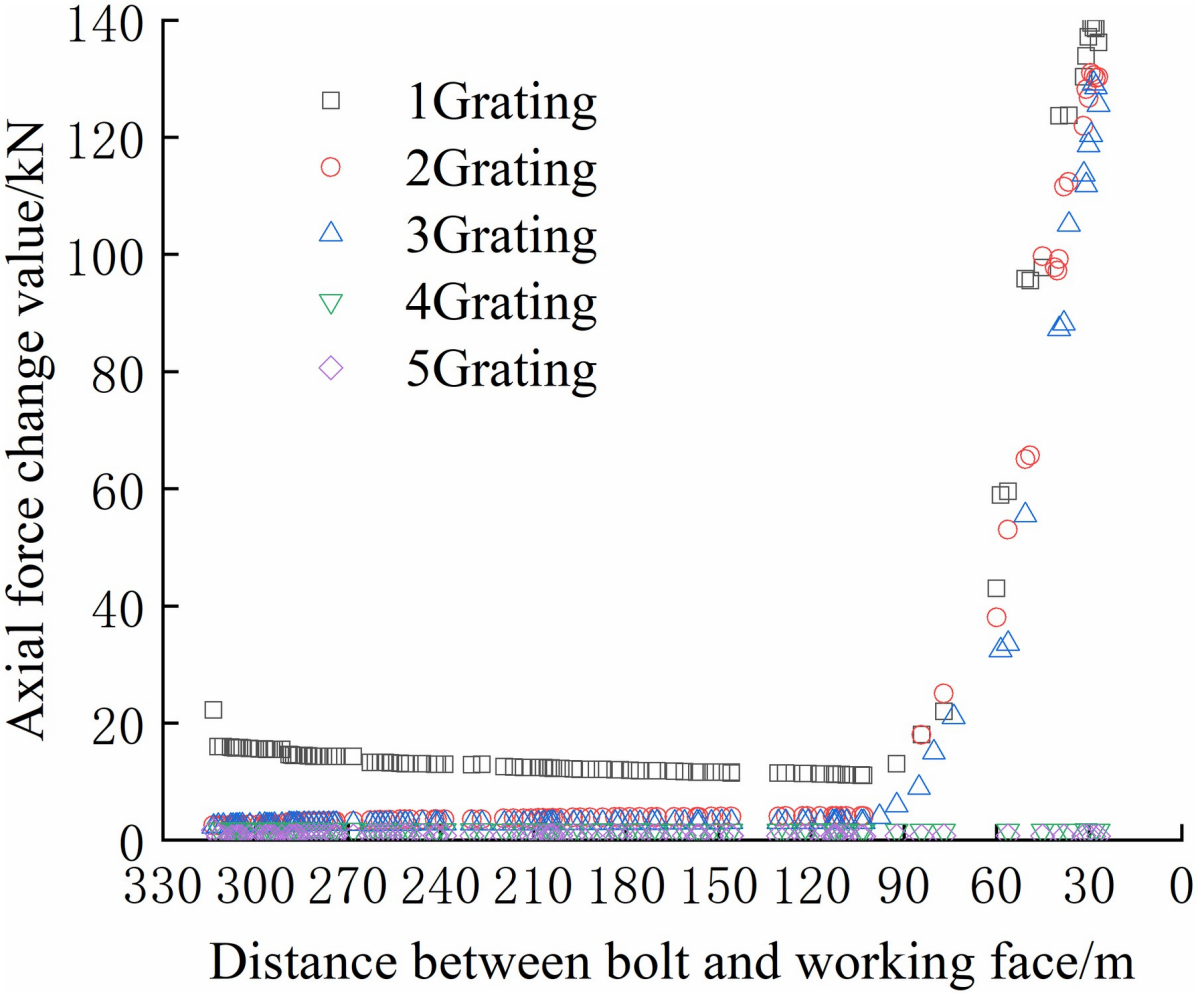
Axial stress monitoring test curve of No.3 bolt.

**Fig 27:**
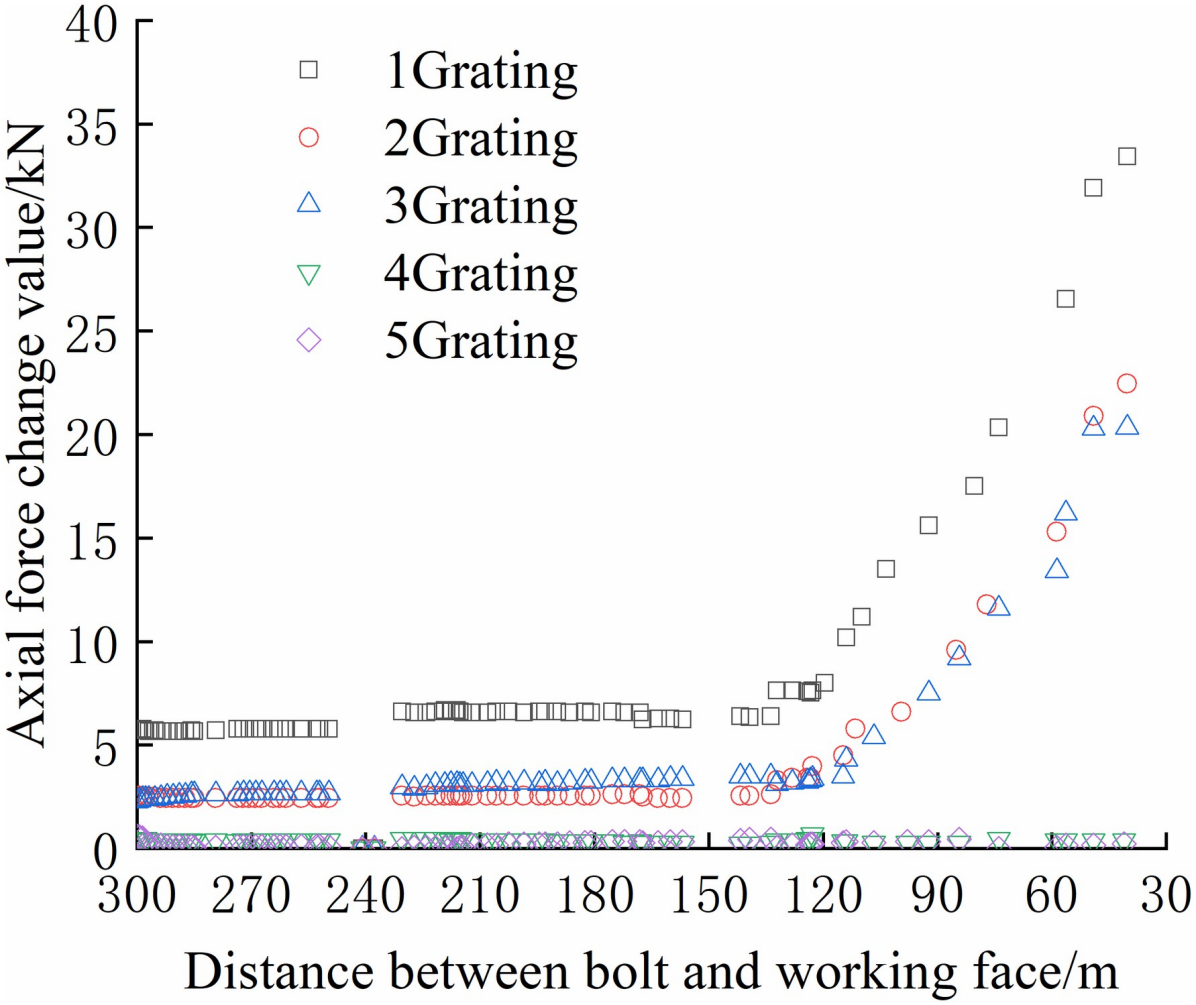
Axial stress monitoring test curve of No.4 bolt.

**Fig 28:**
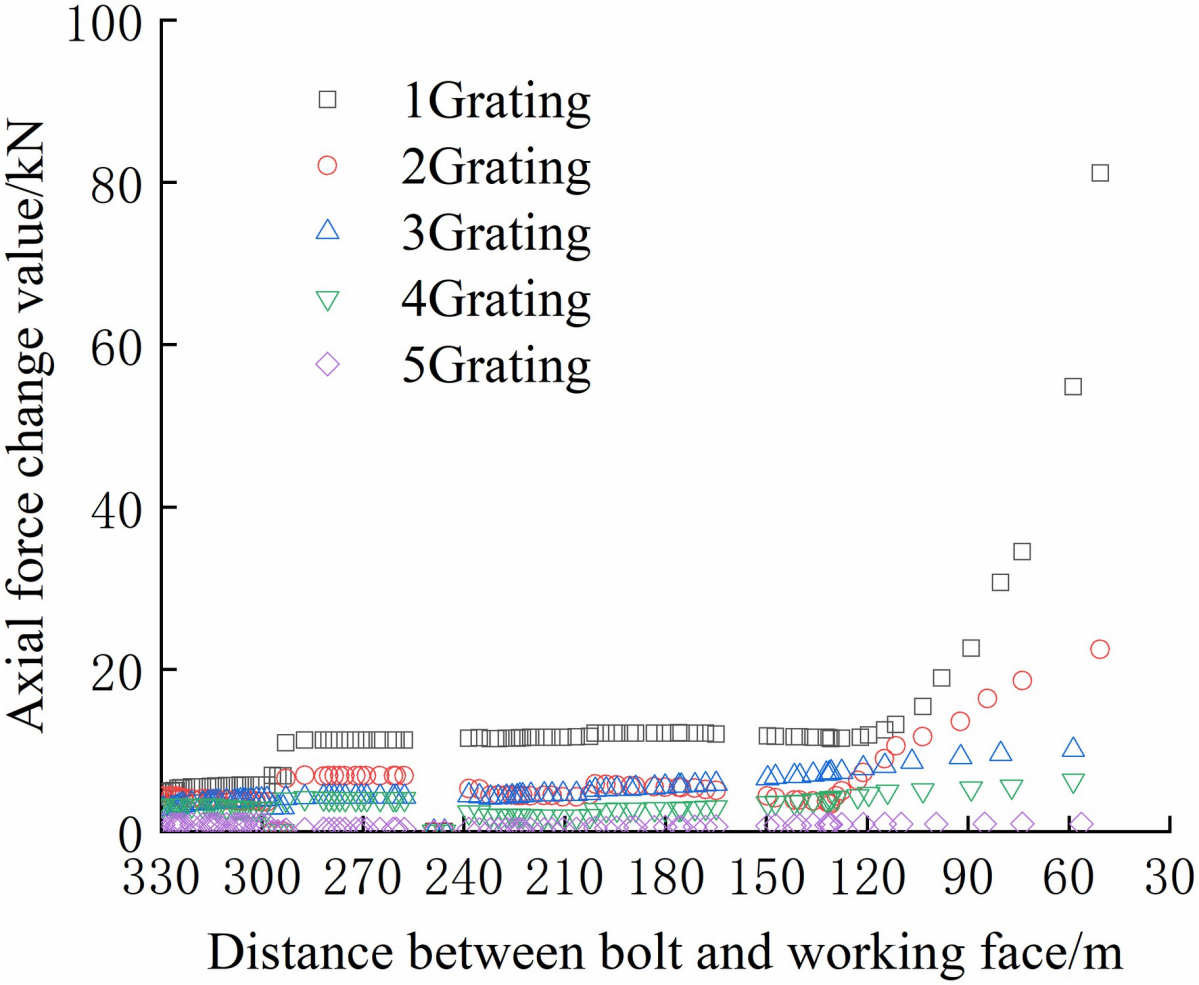
Axial stress monitoring test curve of No.5 bolt.

**Fig 29:**
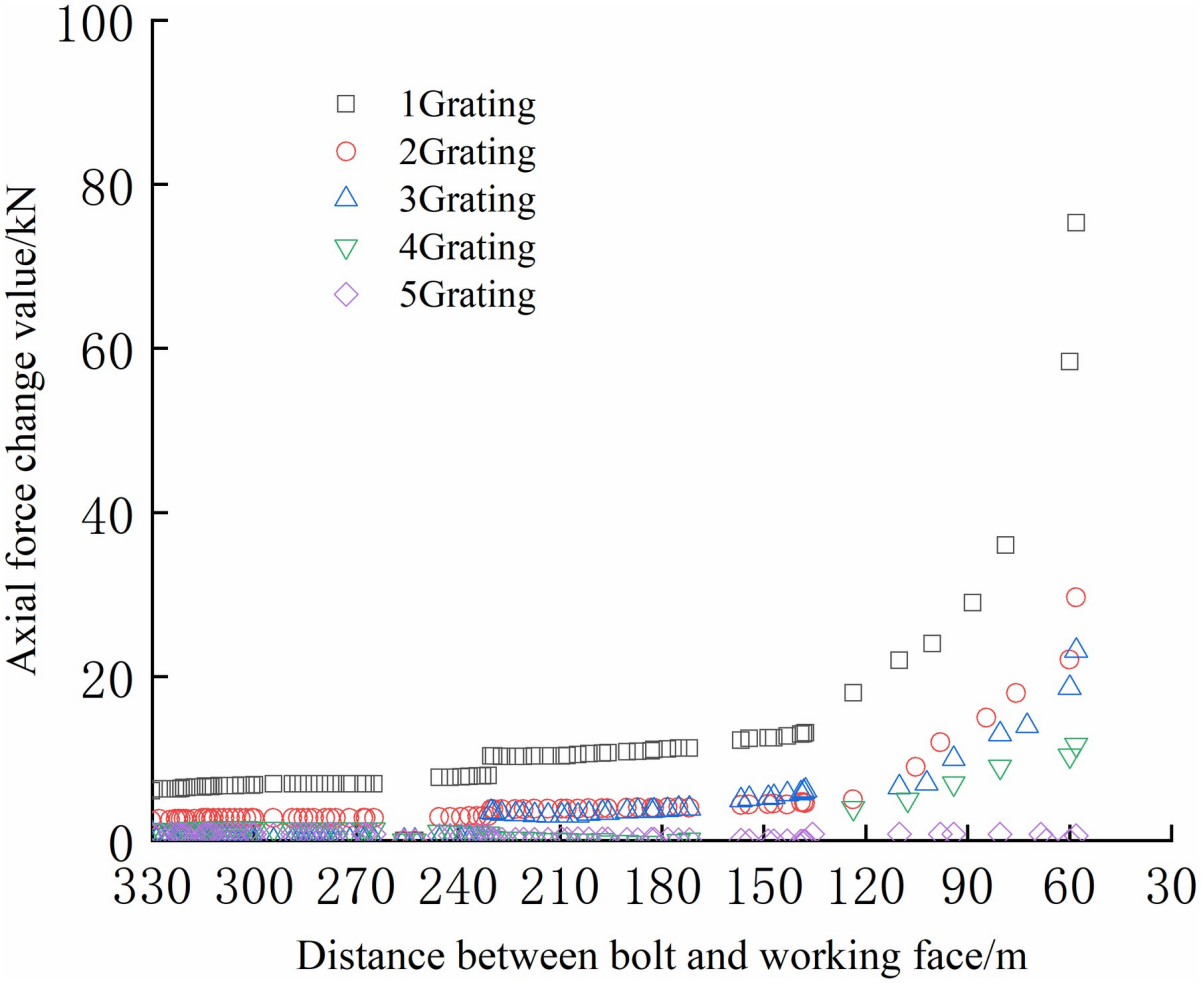
Axial stress monitoring test curve of No.6 bolt.

The FBG sensors were arranged and numbered sequentially at a pitch of 380 mm to facilitate the analysis. The tray section is No.1 grating, and the No. 2, No. 3, and No. 4 gratings are arranged in sequence to the anchoring section, which is No. 5 grating. The data were collected until the No.1 bolt failed to compare and analyze the safety status of each bolt. The axial force of each position can be accurately reflected by the FBG sensor as the working surface advances, as shown in Figs 24–29.

From Figs 24–29, it can be observed that the overall change trend of the axial stress of the positive side bolt is the same, the axial force changes significantly, and the data of No.1 grating changes the most. As the working face advances, it can be observed that the data of the No.1 grating increases faster than the more profound No. 2, No. 3, No. 4, and No. 5. The closer the grating is to the anchoring section, the lower the growth rate of the bolt axial force. The data change in anchoring sections No. 4 and No. 5 was minimal.

The No. 2 and No. 3 bolts were divided into slow growth, rapid growth, and yield. Before the working face was advanced to 60 m away from the bolt, the axial force of the bolt increased slowly. The axial force of the bolt increases rapidly when the working face is advanced to 30–60 m away from the bolt, and the bolt reaches the yielding state when the working face advances to within 30 m from the bolt. Relatively far away from No. 3, No. 4, No. 5, and No. 6 bolts, the axial forces increase slowly and are stable. The axial force data obtained was relatively large only when the working face was advanced to 30 m away from the bolt. The FBG sensor near the pallet can detect a larger axial force than the FBG sensor in the rod of the anchoring section, and its axial force change value is relatively larger.

**Fig 30:**
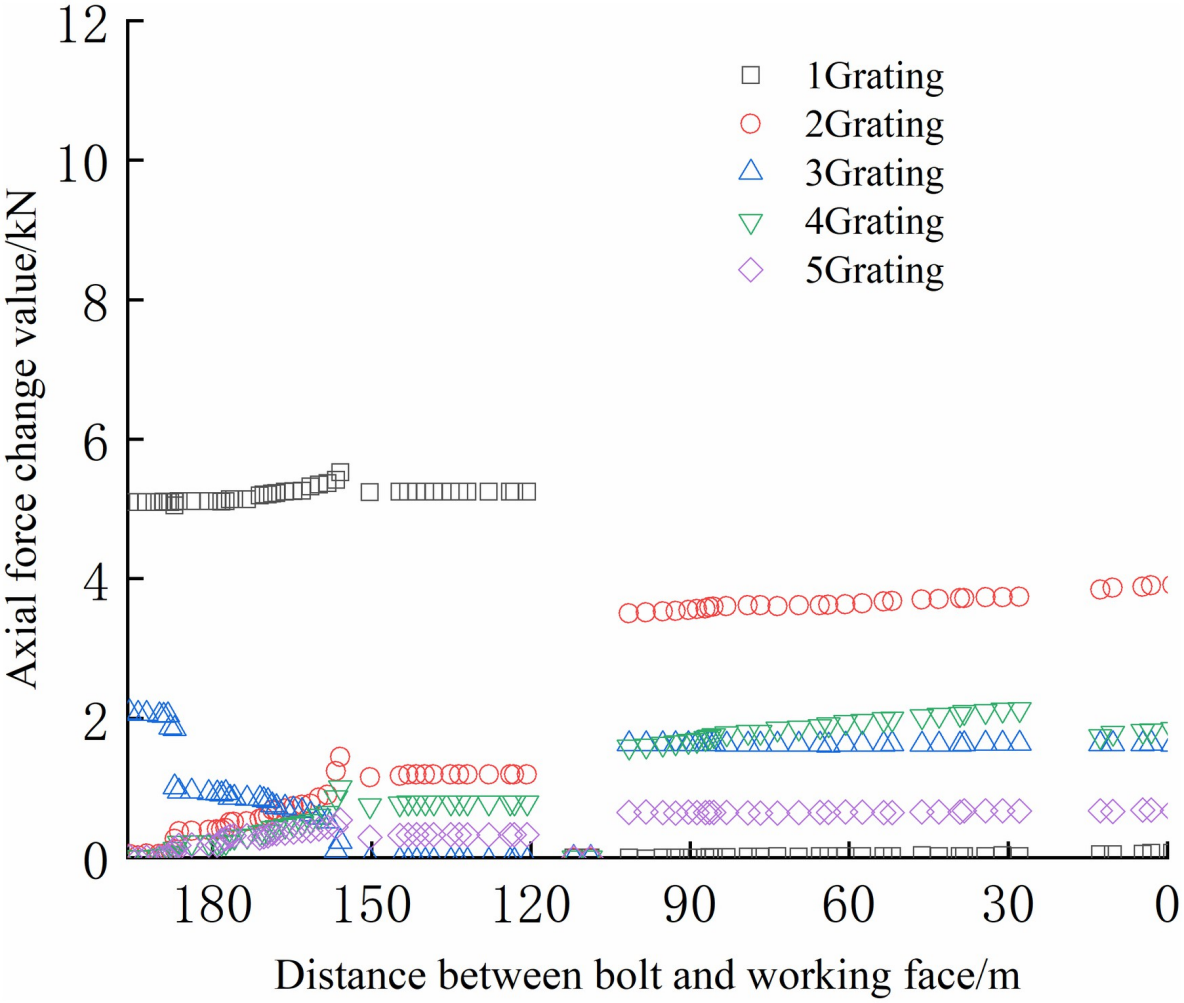
Axial force monitoring test curve of No. 7 bolt.

**Fig 31:**
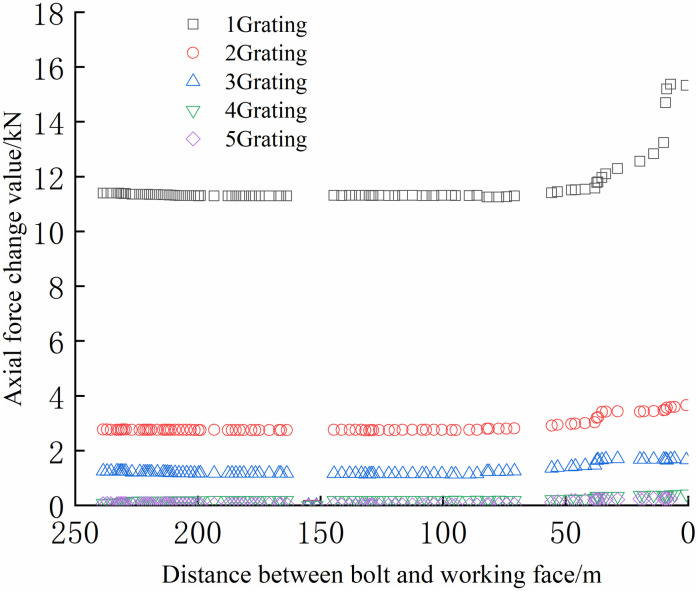
Axial force monitoring test curve of No. 8 bolt.

**Fig 32:**
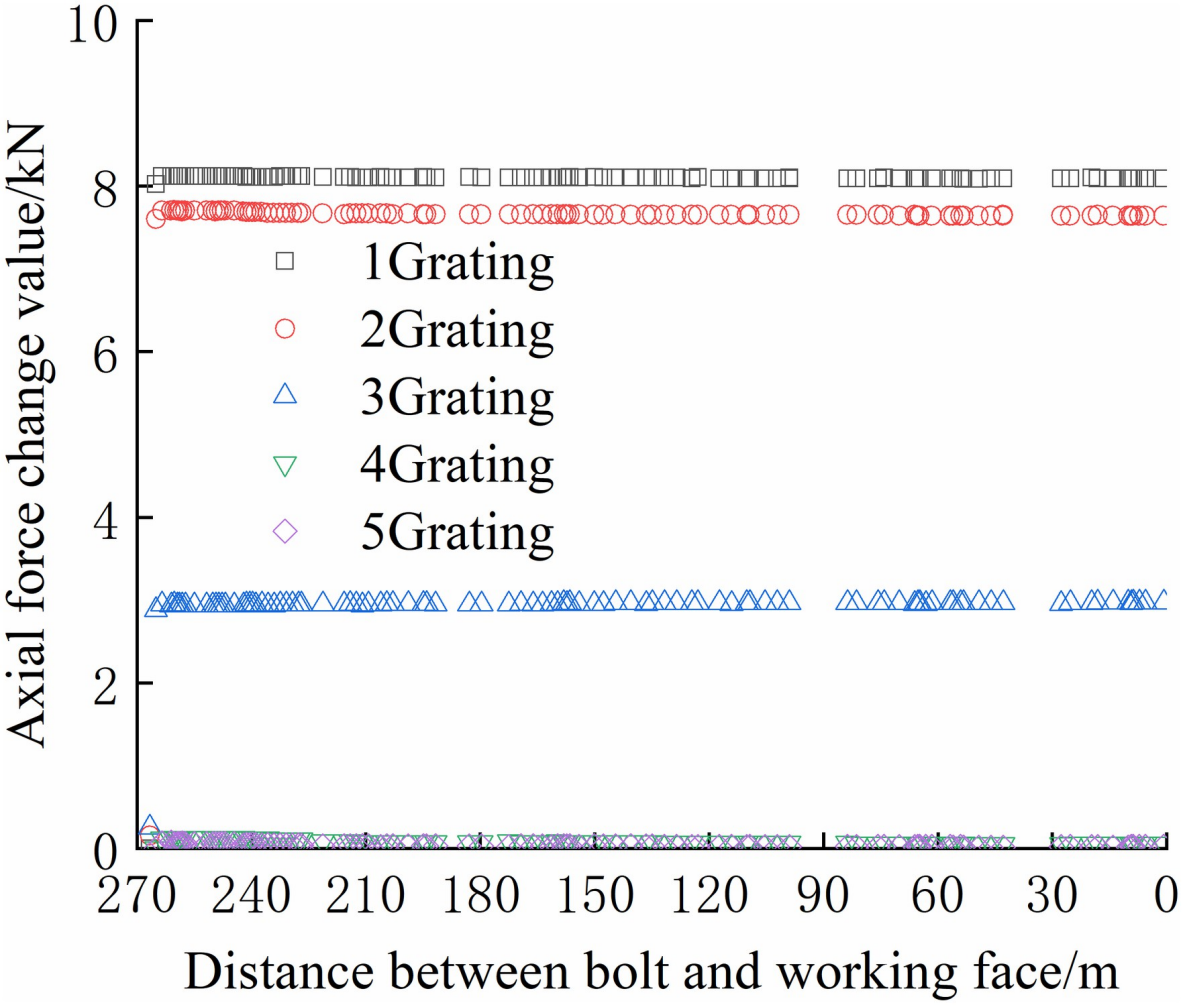
Axial force monitoring test curve of No. 9 bolt.

**Fig 33:**
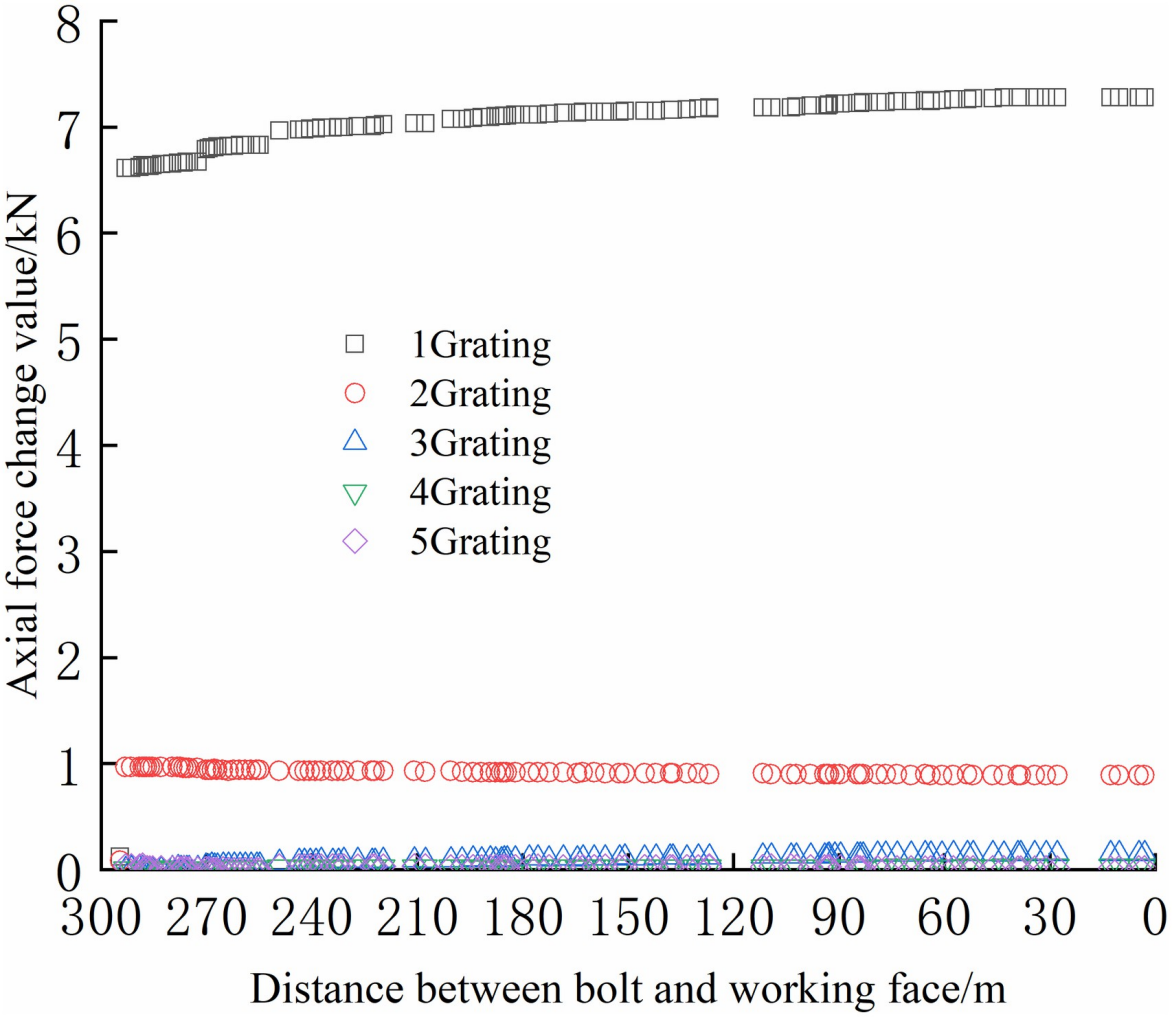
Axial force monitoring test curve of No. 10 bolt.

**Fig 34:**
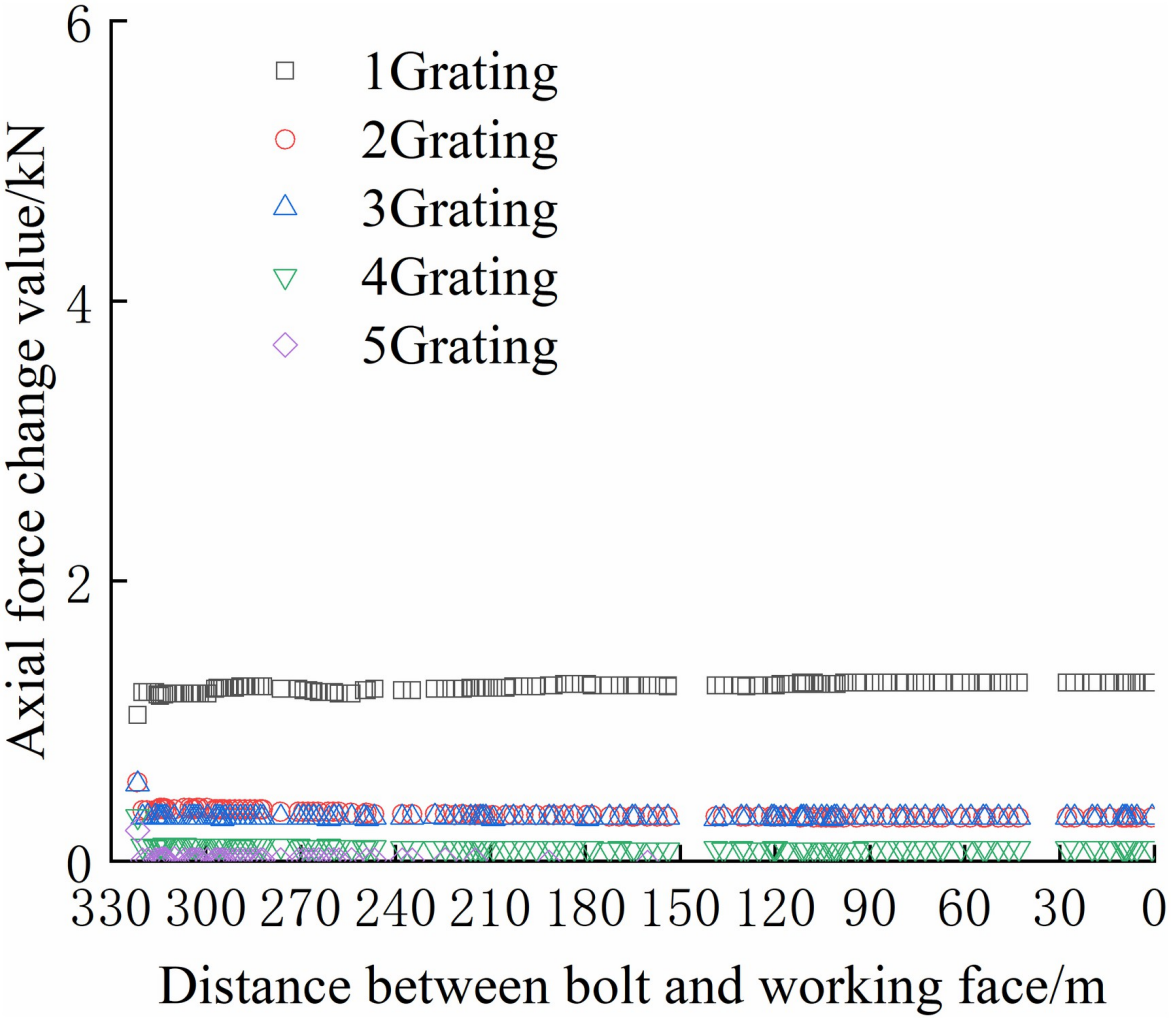
Axial force monitoring test curve of No. 11 bolt.

**Fig 35:**
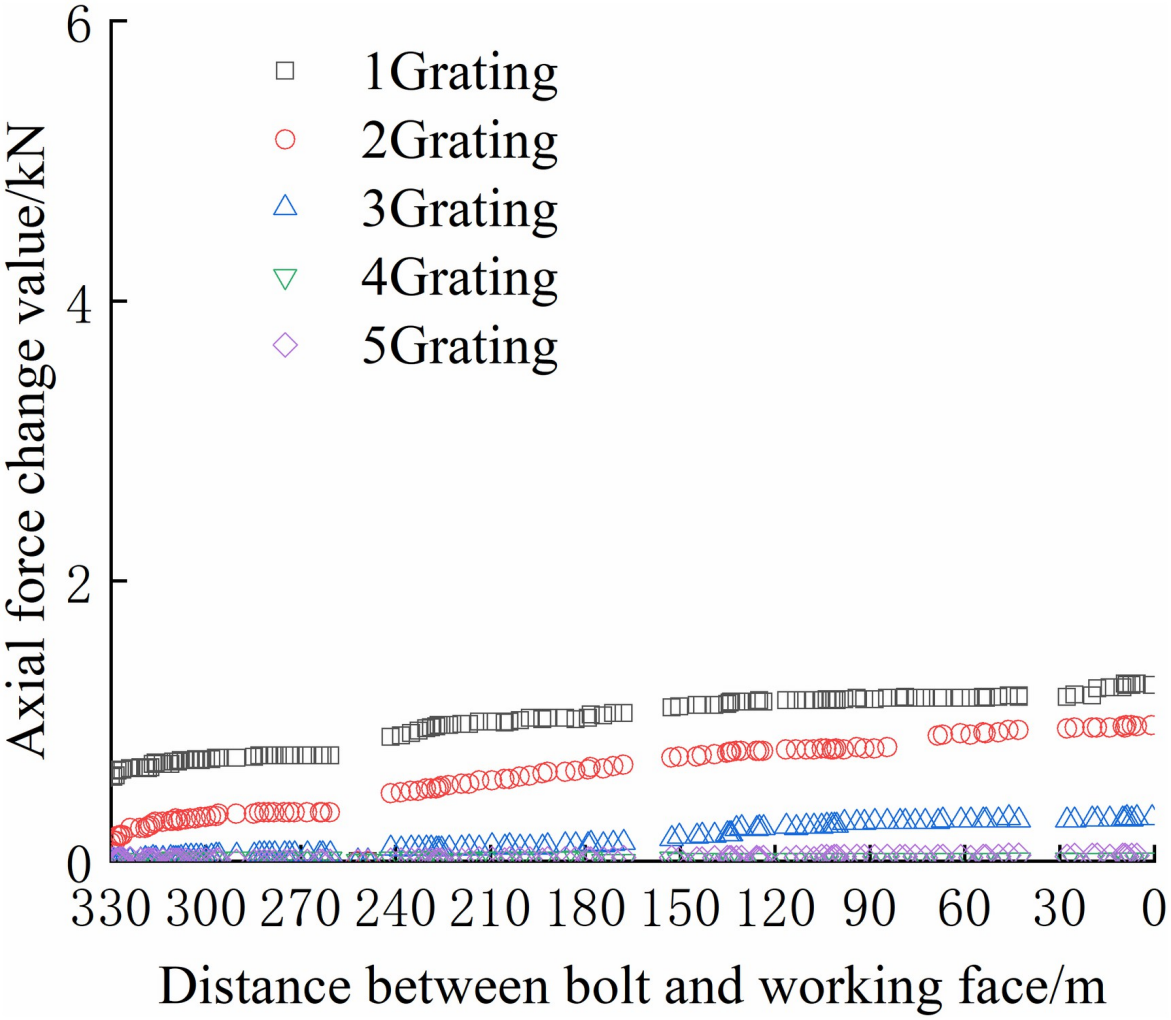
Axial force monitoring test curve of No. 12 bolt.

From Figs 30–35, it can be observed that the overall change trend of the axial stress of the negative side bolt is the same. Although the axial force changes less, it shows an upward trend as the working face advances. Because the No.1 grating is the free section of the bolt, its initial data will be affected by deformation, and thus the axial force of the bolt is greater. The No. 7, No. 8, No. 9, and No. 10 bolts monitor the axial force detected by the FBG sensor near the pallet, are larger than No. 11 and No. 12 bolts, and are more obviously affected by mining. The axial force distribution of the negative bolt was the same as that of the positive bolt. The closer the grating is to the tray, the greater the monitored axial force and the change. Advance support was adopted at 40 m before the positive side of the working face roadway to prevent the two sides from being damaged and the bolt from the bearing part of the rock pressure during the mining of the auxiliary roadway along the channel, which can guard against a significant difference in the axial force of the two sides of the roadway.

### Force characteristics of the bolt body

**Fig 36:**
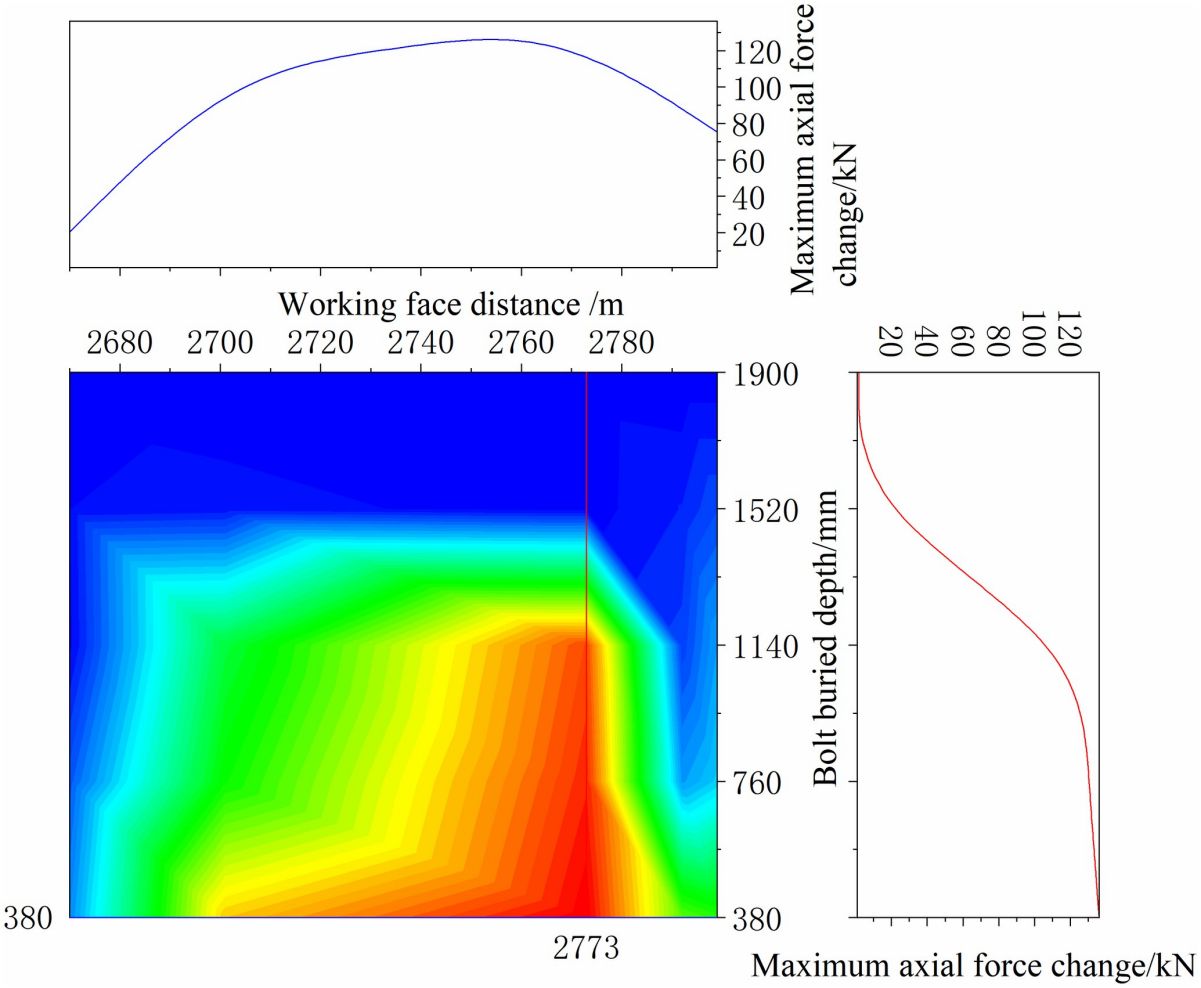
Continuously distributed contour profile curves of bolt monitoring data of positive bolt.

**Fig 37:**
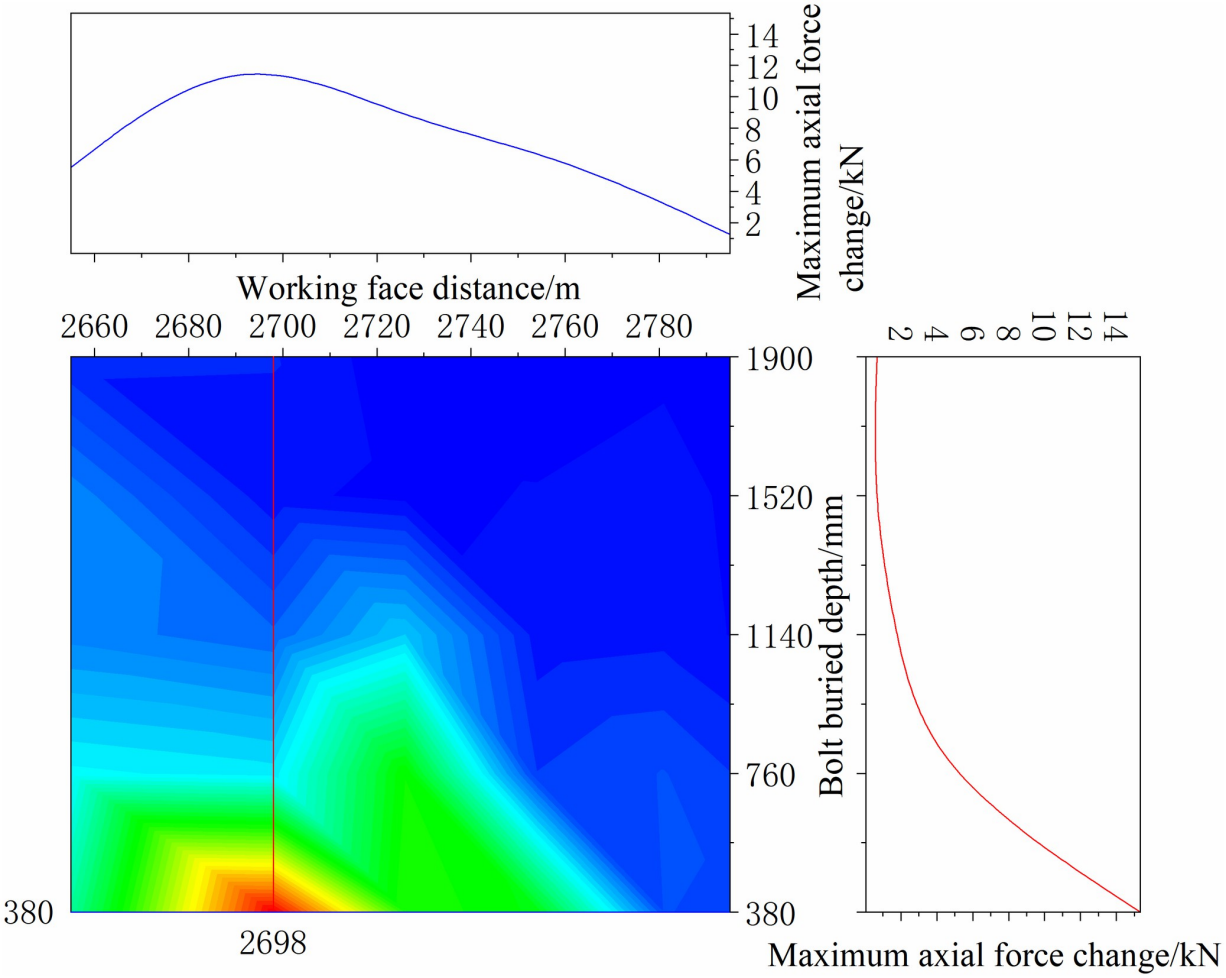
Continuously distributed contour profile curves of bolt monitoring data of negative bolt.

The continuous distribution contour profile curve was obtained according to the monitoring data of the bolt, as shown in Figs 36 and 37.

As shown in Figs 36 and 37, the maximum axial force of the bolt at each position, which was along the direction of the working face and perpendicular to the direction of the working face, was acquired. In the range of 2700–2740 m on the positive side of the mining roadway, the bolt bears a tremendous axial force. Simultaneously, the negative side of the mining roadway in this range bore the maximum axial force. Thus, special attention should be paid to this location when working on the working face.

Along the direction of the working face, because the bolt is buried at a depth of 0–380 mm, it is mainly a free section, and its axial force is not considered. The deeper the bolt is buried, the smaller the maximum axial force of the bolt. Because the bolt is buried deep in the anchoring section, and it is relatively compatible with the surrounding rock and moves with the deformation of the rock, the axial force changes slightly. The bolt is buried shallowly in the free section, and the rod body is close to the tray and can be deformed freely; thus, the axial force changes significantly. Compared with the negative bolts, the axial force of the positive bolts changes more obviously, and the axial force change value is greater. The location of the roadside adjacent to the working face has the shortest distance affected by mining, and the impact of mining is more direct than that of other locations.

### Safety evaluation of bolts

As the working face advances, the top bolt axial force at each position shows a slow growth trend. During the advancement of the working face, the data are not continuous owing to the influence of various factors. Part of the data is only collected at the center wavelength when the working face advances to the closed position of the bolt. When the working face advances, only the axial force of the No. 2 bolt and the No. 3 bolt increased rapidly until yielding. The maximum axial force changes of the bolt are listed in Table 2.

**Table 2:** Maximum axial force change of bolt.

Bolt number	The maximum value of bolt axial force change /kN	The ratio of bolt axial force to design anchoring force /%
No. 1	20.44	75.55
No. 2	108.11	>100
No. 3	136.15	>100
No. 4	33.42	91.78
No. 5	81.13	>100
No. 6	75.29	>100
No. 7	5.52	56.9
No. 8	15.32	69.15
No. 9	8.09	60.11
No. 10	7.27	59.09
No. 11	1.27	51.59
No. 12	1.25	51.56

The basic parameters of the bolt support in GB/T 35056–2018 are shown in Table 3.

**Table 3:** Basic parameters of bolt support.

Parameter name	Unit	Parameter value
Bolt length	m	1.6–3
Nominal diameter of the bolt	mm	16–25
Bolt pre-tightening force	kN	30–60% of the yield force of the bolt
Bolt design anchoring force	kN	The standard value of yield strength of the bolt
Bolt pitch	m	0.6–1.5
Bolt spacing	m	0.6–1.5

According to the Technical Specification for bolt support in GB/T 35056–2018 and [22], bolts can be divided into normal, abnormal, dangerous, and damaged.

Normal state: The bolt’s axial force or deformation is within the allowable design range, and the bolt is in the expected working state of the design. The axial force was less than 60% of the designed anchoring force.

Abnormal state: The bolt is in an abnormal working state, but there are still some safety reserves, and the safety state is overall controllable. The axial force was 60–80% of the designed anchoring force.

Dangerous state: The axial force of the bolt reaches the designed anchoring force, the bolt is in an abnormal working state, the safety reserve is meager, and the safe state is on the verge of losing control. The axial force was 80–100% of the designed anchoring force.

Failure state: The bolt reaches its limit state, and it is destroyed.

Based on the above evaluation methods, the evaluation results for the safety status of the mining roadway bolts are presented in Table 4.

**Table 4:** Bolt safety status statistics.

Bolt status	Condition	Bolt number
Normal	The axial force is less than 60% of the designed anchoring force	No.7, No. 10, No. 11, No. 12
Abnormal	The axial force is less than 60–80% of the designed anchoring force	No. 1, No. 8, No. 9
Dangerous	The axial force is less than 80–100% of the designed anchoring force	No. 4
Failure	The axial force is greater than the design anchoring force	No. 2, No. 3, No. 5, No. 6

## Conclusion

1. The axial force change of the positive side bolt is the most obvious, and the axial force change value is greater than that of the negative side bolt. The roadside position near the working face has the shortest distance affected by mining, and the impact of mining is the most direct.
2. The axial force of the positive bolt increased slowly before the working face was advanced to 60 m away from the bolt. The axial force increases rapidly after the working face advances to 60 m away from the bolt. The negative axial force of the bolt increases slowly with the advancement of the working face.
3. As the anchoring section rod body relatively fits the surrounding rock, the axial force change range is relatively small. The rod near the pallet is located in the free section and can be freely deformed, and the axial force change range is relatively large. The axial force of the bolt is unevenly distributed along the rod body of the free section, and the axial force of the rod body near the pallet was the largest.
4. By continuously distributing contour curves from the monitoring data of working face bolts, comparing the distribution of the maximum bolt axial force at each position, the roadway location that is most affected by mining can be determined. Moreover, bolt support and prevention measures need to be taken to prevent accidents.
5. The safety status evaluation index can be used to evaluate the status of the monitoring bolts based on the ratio of the axial force of the bolts to the designed anchoring force. The No. 2, No. 3, No. 5, and No. 6 bolts reached the failure state. The No. 4 bolt was in a dangerous state. The No. 1, No. 8, and No. 9 bolts were in an abnormal state, whereas No. 7, No. 10, No. 11, and No. 12 bolts were in a normal state.
